# Longitudinal measures of monkey brain structure and activity through adolescence predict cognitive maturation

**DOI:** 10.1038/s41593-025-02076-0

**Published:** 2025-10-27

**Authors:** Junda Zhu, Clément M. Garin, Xue-Lian Qi, Anna Machado, Zhengyang Wang, Suliann Ben Hamed, Terrence R. Stanford, Emilio Salinas, Christopher T. Whitlow, Adam W. Anderson, Xin Maizie Zhou, Finnegan J. Calabro, Beatriz Luna, Christos Constantinidis

**Affiliations:** 1 Program in Neuroscience, Vanderbilt University, Nashville, TN USA; 2 Department of Biomedical Engineering, Vanderbilt University, Nashville, TN USA; 3 Institut des Sciences Cognitives Marc Jeannerod, UMR5229 CNRS Université de Lyon, Bron, France; 4 Department of Translational Neuroscience, Wake Forest University School of Medicine, Winston-Salem, NC USA; 5 Department of Radiology, Wake Forest University School of Medicine, Winston-Salem, NC USA; 6 Department of Psychiatry, University of Pittsburgh, Pittsburgh, PA USA; 7 Department of Ophthalmology and Visual Sciences, Vanderbilt University Medical Center, Nashville, TN USA

**Keywords:** Learning and memory, Development of the nervous system

## Abstract

In humans and other primates, adolescence is associated with improvement in cognitive abilities and with changes in brain structure and connectivity. However, how these changes affect neural activity underlying cognitive performance remains unknown. Here we conducted a multilevel, longitudinal study of monkey adolescent neurocognitive development by tracking behavior, neuronal activity and anatomical imaging measures. The trajectory of prefrontal neural activity accounted well for working memory improvements. Complex aspects of activity changed progressively during adolescence, but even simpler attributes, such as baseline rate and variability, had predictive power over behavior. Trajectories of neural activity and cognitive performance were well predicted by maturation of long-distance white matter tracts linking the frontal lobe with other brain areas but, surprisingly, not by decreases in brain volume and thickness, which underlie cognitive changes in humans. Our results link white matter maturation to neural activity changes in adolescent cognitive development.

## Abstract (web summary)

Working memory improves during adolescent brain development. Zhu et al. tracked monkeys through adolescence, revealing that maturation of white matter tracts and refinement of neural firing patterns sharpen working memory precision.

## Main

Maturation of executive functions including working memory is a hallmark of human cognitive development^1–3^. Delayed response tasks reveal an improvement in performance throughout childhood and adolescence in both precision and reaction time (RT)^4–6^. Although such tasks are simple conceptually, enhancement in their performance proceeds in precise tandem with improvement across a range of other cognitive domains, including response inhibition, task switching and planning, suggesting a domain-general process of cognitive maturation^7^. Age-related cognitive improvement coincides with brain structural changes, including inverted U-shaped trajectories of total brain volume driven by decreasing gray matter volumes in adolescence, while white matter volumes continue to increase into adulthood^8,9^. An overall pattern of decreasing thickness in the prefrontal cortex (PFC) is evident into adulthood^10,11^ and represents restructuring processes, such as pruning of infrequently used synapses^12–14^. Changes in the PFC and its connections with other regions continue to progress late into adolescence^15,16^. Myelination in the underlying white matter providing connectivity between frontal and other regions correlates with development of working memory and other cognitive functions^17,18^. Abnormalities in such processes have been associated with the psychopathology of mental illnesses that emerge at the end of adolescence, such as schizophrenia, mood disorders and substance use disorders^19,20^.

The changes in brain activity that underlie cognitive development through adolescence have been addressed primarily with functional magnetic resonance imaging (fMRI) studies, which reveal distinct differences in prefrontal activity patterns between childhood and adulthood in humans during working memory tasks^21,22^. More direct evidence about changes in activity of single neurons and populations has been obtained from nonhuman primate models of adolescence^23,24^. Rhesus macaques (*Macaca mulatta*) enter puberty at approximately 3.5 years of age and reach full sexual maturity at age 5 years, aging at a rate of approximately three times faster than humans^25,26^. Anatomical studies suggest a protracted period of prefrontal cortical development that parallels that of humans^27–29^. Cortical thickness, surface area and white matter myelination also follow similar trajectories to those of humans, at least in childhood^30,31^. Biochemical and anatomical changes have also been characterized in the monkey PFC from prepuberty to adulthood, including changes of interneuron morphology and connections^32,33^.

However, a direct link between cognitive performance in terms of underlying changes in neural activity and concomitant structural brain changes has been lacking. Therefore, we were motivated to track behavior, neuronal activity and anatomical imaging measures at multiple developmental stages with key developmental milestones in a cohort of developing monkeys. By determining the relationship of such measurements, we identified the brain mechanisms that can account for the cognitive improvement seen during this critical period of life.

## Results

**Fig. 1:**
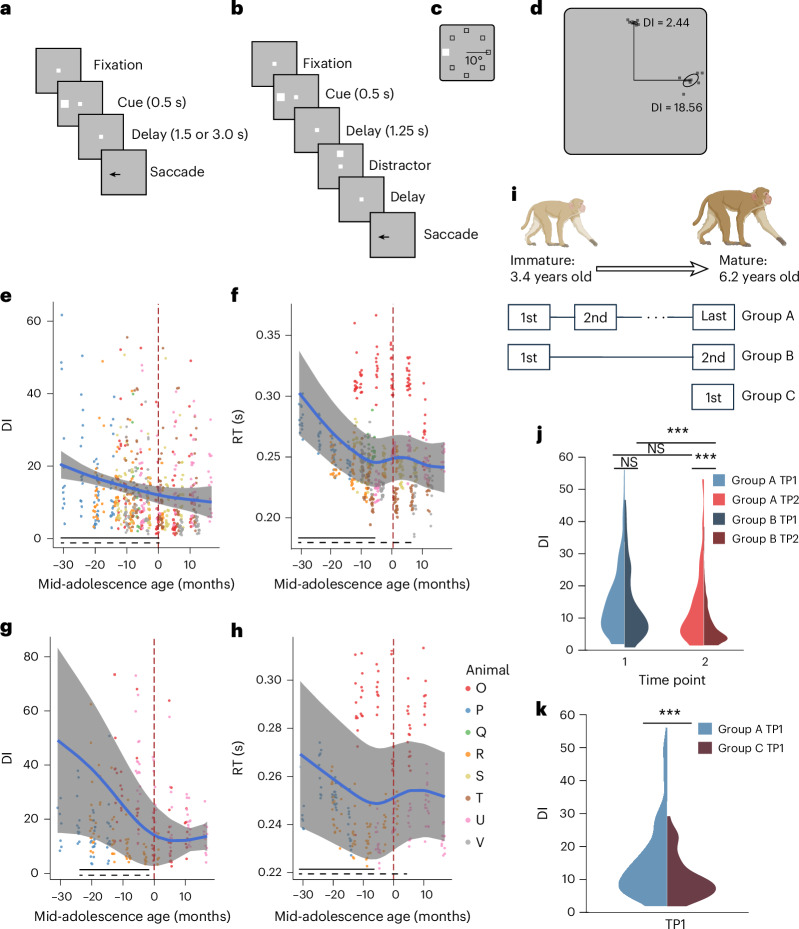
Saccade precision and latency improve during adolescence. **a**, Sequence of events in the ODR task. The monkey is required to maintain fixation while a cue stimulus is presented and after a delay period, when the fixation point turns off, saccade to the remembered location of the cue. **b**, Sequence of events in the ODR with distractor task. After the delay period, a distractor stimulus appears, which needs to be ignored. The monkey is still required to saccade to the remembered location of the cue. **c**, Possible locations of the stimulus presentation on the screen. **d**, Schematic illustration of variability of two groups of saccades. The gray dots represent the endpoints of individual saccades for two stimulus locations. DI, defined as the area within one s.d. from the average landing position of each target is shown. **e**, DI in the ODR task, during the neural recording sessions. Each dot is one session; data from different monkeys are shown in different colors. The blue line shows the GAMM-fitted trajectory. The gray shaded regions denote the 95% confidence intervals (CIs). The dashed vertical line denotes a mid-adolescence age of 0. The horizontal dashed bar denotes significant developmental effect intervals. The horizontal solid bar denotes intervals with significant monotonic developmental effect. **f**, As in **e**, for the RT of saccade in the ODR task. **g**, As in **e**, for the DI in the ODR with distractor task. **h**, As in **f**, for the RT in the ODR with distractor task. **i**, Schematic diagram of the three cohorts of monkeys (groups A–C) used to evaluate behavioral improvement. The image of the monkeys was created with BioRender. **j**, DI in the ODR task of groups A and B at the TP1 and TP2 time points. The violin plot shows the distribution of DI values for both groups at two distinct time points, with the width of the plot indicating the density of the data points. Statistical comparisons were performed with a two-sided, two-sample *t*-test (no adjustment for multiple comparisons). TP1: *P* = 0.21; TP2: *P* = 2.83 × 10^−4^. **k**, DI in the ODR task of groups A and C at the first time point. Two-sided, two-sample *t*-test (no adjustment for multiple comparisons): *P* = 9.35 × 10^−5^. ***P < 0.0001. Source data

**Extended Data Fig. 1:**
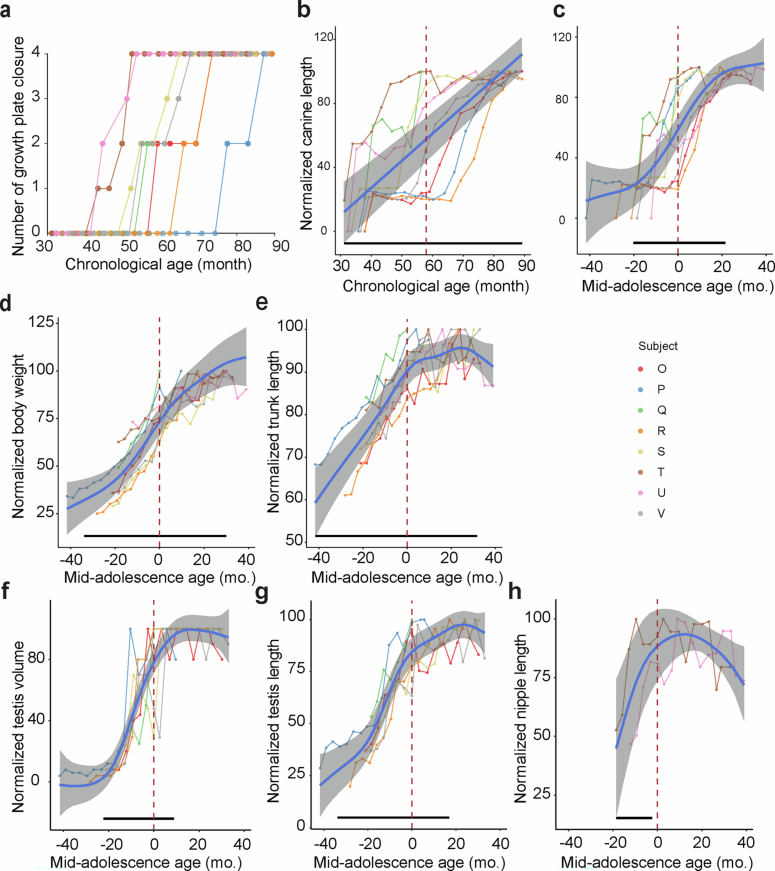
Developmental profile and mid-adolescence age. (**a**) Number of growth plate closure of each monkey as a function of chronological age. (**b**) Average canine length of the same subjects, aligned on their chronological age. Blue line showed the GAMM fitted trajectory. (**c**) same as in (**b**) but aligned on their mid-adolescence age. (**d** and **e**) Body weight and trunk length, presented in the same fashion as in (**c**). (**f** and **g**) Testis volume and size (length of longer dimension) of the male monkeys, presented in the same fashion as in (**c**). (**h**) Nipple length of the female monkeys, presented in the same fashion as in (**c**). In each plot the dashed vertical line denotes the mid-adolescence age 0. The horizontal bar denotes significant developmental effect intervals. Gray shaded regions denote the 95% confidence intervals (CIs) around the fitted mean values. Source data

We tracked developmental measures longitudinally in a cohort of eight monkeys (group A in Fig. 1; six males, two females) every 3 months, in tandem with neurophysiological recordings and MRI, from an age of 3.0 ± 0.1 to 7.1 ± 0.1 years (corresponding to human ages of ~9–21 years). Another six older monkeys matched for training time in the task at different time points were used for control comparisons (groups B and C). At the beginning of the study, morphometric measures were all consistent with individuals in a growth trajectory; however, the emergence of developmental markers and secondary sexual characteristics, such as the eruption of canines, varied considerably between individuals (Extended Data Fig. 1). This reflects the variability in pubertal timing, which defines the period of adolescence. Therefore, we sought to align individual growth trajectories on a biological developmental marker rather than chronological age and relied on the closure of the epiphyseal growth plate, a well-established indicator of skeletal maturation in humans^34,35^. Thus, we defined a ‘mid-adolescence’ age for each monkey as the time of tibial epiphyseal closure (Methods). We also examined the developmental trajectory for each morphometric measure on chronological and mid-adolescence age independently using nonlinear regression models (generalized additive mixed models (GAMMs)). We compared models aligned on chronological and mid-adolescence age for each of seven morphometric measures using the Akaike Information Criterion. All but one morphometric measure had a lower Akaike Information Criterion when using mid-adolescence age, compared with using chronological age (Supplementary Table 1). For example, plotting canine length as a function of age across individuals generated a deceptively monotonic curve throughout adolescence and into adulthood (Extended Data Fig. 1b). Instead, aligning on mid-adolescence age revealed that lengthening of canines was completed 21.7 months after the mid-adolescence age on average (Extended Data Fig. 1c). Thus, mid-adolescence age provided a more accurate representation of each animal’s developmental stage compared to their chronological age, allowing us to effectively account for the individual variability and more directly examine the effects of maturation (Extended Data Fig. 1b,c). The mean mid-adolescence age across individuals was 57.9 ± 3.6 months (corresponding to a human age of ~14.5 years). All morphometric measures exhibited significant development-related differences and followed a similar nonlinear developmental trajectory, with rapid changes before and around mid-adolescence age, before plateauing when adulthood was reached (Extended Data Fig. 1c–h).

### Working memory performance and developmental measures follow a common trajectory

We evaluated working memory performance with variants of the oculomotor delayed response (ODR) task (Fig. 1a–c), which has been extensively used in human studies^4,7^. Monkeys were required to observe a visual cue that could appear at one of eight locations and, after a delay period, to make an eye movement to the location of the remembered visual stimulus. A distractor stimulus appeared in a variant of the task (ODR + distractor). Behavioral performance was collected at time points spaced ~4 months apart from 3.4 to 6.2 years old (Supplementary Fig. 1). Animals were able to perform the task from their earliest time point and achieved an average of 79% correct responses (excluding trials aborted before the end of the delay period; Supplementary Fig. 2), in agreement with previous studies in monkeys^23^ and human children^5^. Human studies revealed that the precision of working memory improves in adolescence^4,5,7^. We similarly sought to capture changes in working memory functions beyond simply task performance.

Therefore, we used the dispersion index (DI) (Fig. 1d) to quantify the precision of saccadic endpoints (Methods) and relied on GAMMs to examine development-related effects. Like prior findings in humans using the same task^36^, working memory precision improved with maturation (GAMM, *F*_(1, 568)_ = 3.31, *P* = 0.0002; Fig. 1e). Additionally, we examined response latency by calculating the RT of each trial. Importantly, RT was faster with maturation, indicating that improved precision was not achieved because of a speed–accuracy trade-off (GAMM: *F*_(1, 568)_ = 7.76, *P* = 1.41 × 10^−6^; Fig. 1f). As substantial variability in precision and RT was present between individuals, we used a random effect to capture individual differences and ensure that the trajectory shape was not dominated by a single monkey. Repeating the analysis by leaving one monkey out also revealed very similar results (Supplementary Tables 2 and 3).

Working memory precision and latency also improved with age for the variation of the working memory task involving a distractor (DI GAMM: *F*_(1, 238)_ = 2.09, *P* = 0.0001; RT GAMM: *F*_(1, 238)_ = 5.96, *P* = 3.47 × 10^−5^; Fig. 1g,h). In both tasks, significant developmental improvements occurred in early adolescence (−31 to 0 months relative to the mid-adolescence age). RT had significant growth rates throughout adolescence (−31 to −7 months relative to the mid-adolescence age), with greater changes earlier. In general, rapid and large developmental improvements occurred before and around the mid-adolescence age of monkeys for both saccade precision and latency, followed by a slowdown of change and plateau in late adolescence and early adulthood.

Monkeys tested later in development had more cumulative exposure to the task than in early age, as an inevitable consequence of our longitudinal experimental design. To evaluate the effect of exposure to the task, we compared the behavioral performance of this group of animals (group A) with the performance of two other groups of animals following similar training and developmental procedures (Fig. 1i). We reanalyzed a group with four animals (group B, all males) that was introduced to the same task at a similar starting age (median = 4.3 years) and were trained under the same protocol^23^. After completing their first time point (TP1) (young stage), their second time point (TP2) for behavioral testing began 1.6–2.1 years later. We compared saccade precision and its changes in the two groups of animals during their respective first and second testing time points. Group B animals had a slightly lower DI than group A animals during the first testing time point, which did not reach statistical significance considering that they are slightly older than group A monkeys (mean DI of group A = 15.36; mean DI of group B = 13.66; *P* = 0.21, two-sample *t*-test; Fig. 1j). When comparing the DI at the second time point, we saw a significant difference in saccade precisions between the groups even though the two groups had comparable exposure to the task (mean DI of group A = 13.15; mean DI of group B = 7.59; *P* = 2.83 × 10^−4^, two-sample *t*-test; Fig. 1j). The result indicates that the difference of age during the two testing time points in the two groups accounted for a significant difference in saccade precisions at the second time points. Next, we proceeded to train and test a third group (group C) of two animals, which were first trained to perform the task as adults (6.5 years old), with the same training procedure. Adult animals achieved a significantly lower DI in the ODR task despite having the same training exposure as young animals during their first testing time point (mean DI of group A = 15.36; mean DI of group C = 10.54; *P* = 9.35 × 10^−5^, two-sample *t*-test; Fig. 1k). These results indicate that development confers an improvement in cognitive function and that this cannot be accounted for solely by cumulative task experience.

### Reliability of firing metrics of PFC neurons improves with adolescent development

**Fig. 2:**
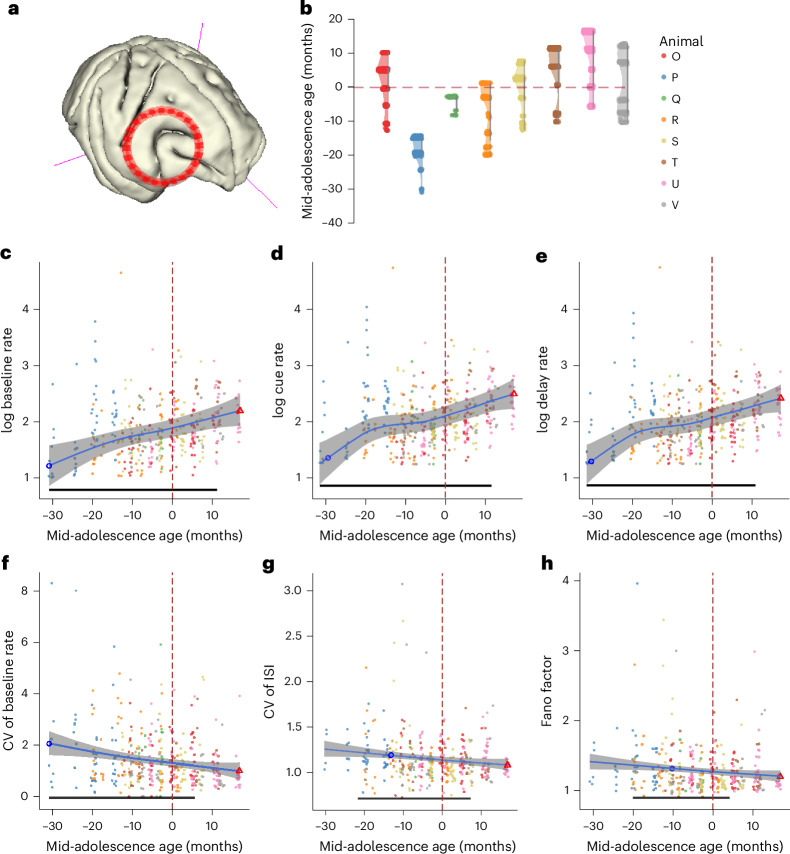
Neurophysiological recordings and neuron firing metrics. **a**, Reconstructed MRI T1 image of one monkey’s brain, with placement of the recording chamber indicated. **b**, Distribution of neurons recorded across time points. Each point represents a neuron at its recorded time relative to mid-adolescence age shown on the *y* axis. Each vertical distribution represents one monkey. **c**–**e**, log-normal average baseline (**c**), cue (**d**) and delay period (**e**) firing rate of neurons in each session recorded during the ODR task. Each dot is one session. The blue line shows the GAMM-fitted trajectory. The gray shaded regions denote the 95% CIs. The blue circle denotes the time of peak development velocity. The red triangle denotes the time of maximum or minimum value. The black bar indicates regions of significant development-related change. **f**–**h**, CV of firing rate (**f**), CV of ISIs (**g**) and Fano factor of neurons (**h**) in each session recorded during the ODR task. Each dot represents the mean value from one recording session. The blue line shows the GAMM-fitted trajectory; the gray shaded regions denote the 95% CIs. The dashed vertical line denotes the mid-adolescence age of 0. The horizontal bar denotes significant developmental effect intervals. Source data

**Extended Data Fig. 2:**
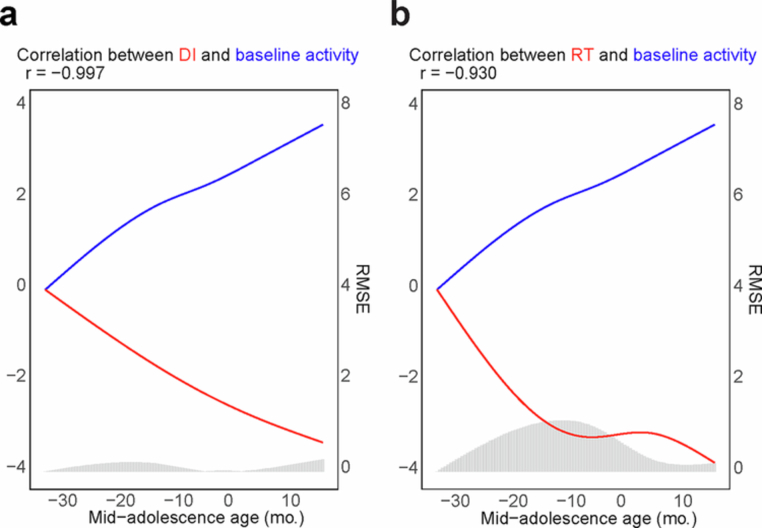
Correlation between the GAMM fitted trajectories of behavioral measures and baseline activity of PFC neurons. (**a**) Correlation between the GAMM fitted trajectories of DI and baseline activity of PFC neurons. Curves represent normalized GAMM fitted trajectories. Histogram indicates RMSE between corresponding prediction values of the GAMMs. (**b**) As in (**a**), for the GAMM fitted trajectories of RT and baseline activity of PFC neurons.

Neurophysiological recordings were made in areas 46 and 8a of the dorsolateral PFC (Fig. 2a) from each animal. To make an unbiased comparison of PFC neural activity, we recorded all neurons that we encountered. For each neuron, we determined an age relative to the mid-adolescence age of the animal at the day the neuron was recorded. In total, our database contained 2,131 neurons from 387 sessions across eight animals (Supplementary Table 4), covering an extended range of adolescence (Fig. 2b and Supplementary Fig. 1). We first determined what aspects of the PFC firing rate change over the course of development that could account for the changes we observed in working memory task performance. PFC activity during baseline (before the cue) fixation, visual response (cue) and delay epoch changed along with maturation; each followed a trajectory similar to the common trajectory that body growth and cognitive function shared, which is characterized by a significant increase early on, continuing to increase after the mid-adolescence age (fixation period (GAMM): *F*_(1, 378)_ = 5.9, *P* = 3.4 × 10^−6^; significant developmental improvements −31 to 11 months relative to mid-adolescence age (Fig. 2c); cue period (GAMM): *F*_(1, 378)_ = 4.4, *P* = 0.00023; significant developmental improvements −31 to 11 months relative to mid-adolescence age (Fig. 2d); delay period (GAMM): *F*_(1, 378)_ = 4.7, *P* = 0.00020; significant developmental improvements −31 to 11 months relative to mid-adolescence age (Fig. 2e)). We computed the correlation between the GAMM-fitted trajectories of behavioral measures and baseline activity of PFC neurons, which revealed a remarkable correlation of neural activity with saccadic precision (DI versus baseline activity: *r* = −0.997; permutation test, *P* < 0.0001; Extended Data Fig. 2a) and also strong correlation with RT (RT versus baseline activity: *r* = −0.930; permutation test, *P* < 0.0001; Extended Data Fig. 2b).

As improvement in behavior was to a large extent a decrease in variability, we sought to test whether measures of neural firing variability also declined. Therefore, we calculated the coefficient of variation (CV) of the inter-spike interval (ISI) and the CV of firing rate across trials during pre-cue fixation, which represents the baseline activity, to investigate the intrinsic firing properties of neurons at different developmental stages. Both the CV of ISI and the CV of baseline firing rate significantly decreased before stabilizing at the adult level (CV of firing rate (GAMM): *F*_(1, 378)_ = 3.68, *P* = 0.00014; significant developmental improvements −31 to 6 months relative to mid-adolescence age (Fig. 2f); CV of ISI (GAMM): *F*_(1, 378)_ = 2.16, *P* = 0.002; significant developmental improvements −21 to 7 months relative to mid-adolescence age (Fig. 2g)). Similarly, we tested the Fano factor of spike counts. Overall, Fano factor values were significantly lower in the adult than in the young PFC (Fig. 2h); changes along the maturation resembled the trajectory observed in firing rates (GAMM: *F*_(1, 326)_ = 1.8, *P* = 0.0049; significant developmental improvements −19 to 5 months relative to mid-adolescence age). Repeating the analysis of neural activity metrics by leaving one monkey out also revealed very similar results (Supplementary Tables 5–7).

Other aspects of neuronal firing were relatively stable in adolescence. These included the width of tuning curves for the eight cue locations measured during the cue and delay periods. Tuning width did not show significant changes during adolescence in either the cue (GAMM: *P* = 0.109) or delay (GAMM: *P* = 0.271) period. The result did not change after eliminating neurons with a low firing rate (baseline: ≤0.5 Hz) and poor Gaussian fitting (*R*^2^ < 0.5) (cue: *P* = 0.273, GAMM in Supplementary Fig. 3a; delay: *P* = 0.091, GAMM in Supplementary Fig. 3b). We also quantified the amount of information carried in single neurons about the location of the cue. We used the percentage of explained variance (*ω*PEV) statistic to measure the extent to which the variability in neural firing rate in different trials was explained by cue location during stimulus presentation. Overall, PFC neurons were selective for cue location during the cue (session average: *ω*^2^ = 0.034; permutation test *P* = 0.003) and delay (session average: *ω*^2^ = 0.033, *P* = 0.012) period. Next, we examined the developmental effect on single-neuron stimulus selectivity. *ω*PEV during visual cue or memory delay did not change significantly during adolescence (*P* = 0.995; Supplementary Fig. 3c). We assessed whether this averaged measure is driven by subpopulations of neurons that are task-responsive. Task-responsive neurons had higher *ω*PEV than the whole population, but there was no significant development effect on *ω*PEV during visual cue (*P* = 0.065 (GAMM)) or memory delay (*P* = 0.372 (GAMM)). We additionally tested whether the results for the whole population were affected by neurons with a low firing rate during certain task epochs. The result did not change even after eliminating neurons with a low mean firing rate in each epoch (≤ 0.5 Hz). The intrinsic timescale is another fundamental property that reflects how quickly a neuron responds to changes in input or internal states. The intrinsic timescales remained stable during the adolescent period (*P* = 0.227 (GAMM); Supplementary Fig. 3d).

### The dimensionality of PFC responses increases in adolescence

**Fig. 3:**
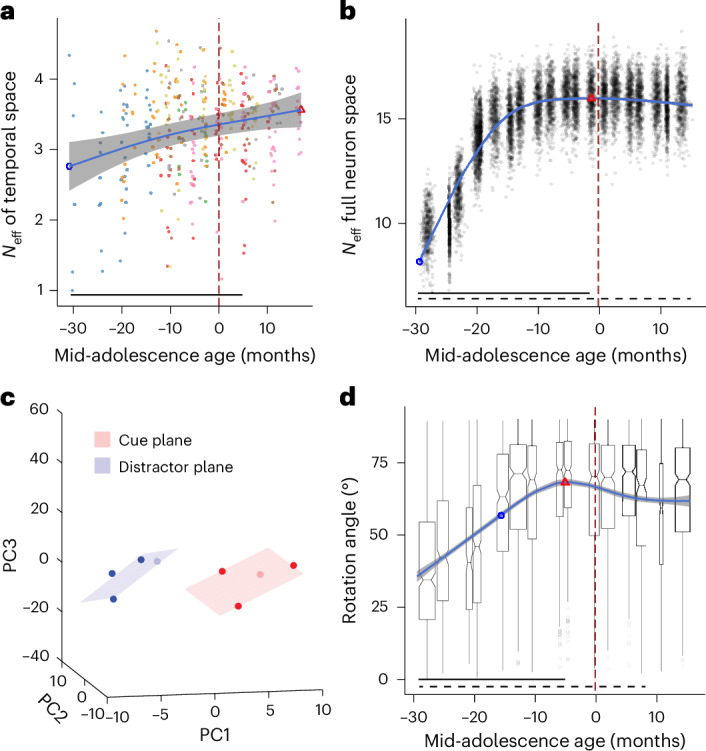
Neural dimensionality and subspace rotation as a function of mid-adolescence age. **a**, Effective temporal dimensionality as a function of age. Each dot is the *N*_eff_ estimated in a single neuron. The blue line shows the GAMM-fitted trajectory. The gray shaded regions denote the 95% CIs. The blue circle denotes the time of peak development velocity. The red triangle denotes the time of maximum or minimum value. The dashed vertical line denotes the mid-adolescence age as time 0. The horizontal bar denotes intervals with significant developmental effect. **b**, Full neural dimensionality. The scatter plot shows the distribution of the dimensionality of the full neural response during the stimulus presentation and delay epochs. Each dot is the estimated effective dimensionality of a pseudo-population of neurons at the age the neurons were recorded (Methods). The blue line shows the GAMM-fitted trajectory. The gray shaded regions denote the 95% CIs. The blue circle denotes the time of peak development velocity. The red triangle denotes the time of maximum dimensionality. The dashed vertical line denotes the mid-adolescence age of 0. The horizontal bar denotes significant developmental effect intervals. **c**, Example of low-dimensional representation of spatial stimuli during the cue and distractor epochs in correct trials in ODR with distractor task (population mid-adolescence age = −20 month). Rotation *φ* = 44°. **d**, Rotation angle between cue and distractor subspace as a function of age. The box plot summarizes the distribution of rotation angles estimated from pseudo-populations of neurons at their respective average ages in each interval (Methods). The box plot indicates the median (center line), the 25th–75th percentiles (box bounds) and the minima and maxima within 1.5 times the interquartile range (whiskers). The blue line shows the GAMM-fitted trajectory. The gray shaded regions denote the 95% CIs (*n* = 30 neurons per interval for 15 intervals, total *n* = 450 neurons from four monkeys). The blue circle denotes the time of peak development velocity. The red triangle denotes the time of maximum rotation angle. The dashed vertical line denotes the mid-adolescence age of 0. The horizontal bar denotes significant developmental effect intervals. Rotation angles were obtained using bootstrap resampling (500 replicates per interval for a total = 7,500 values). Source data

The measures of neural activity examined so far relied on mean values computed over entire task epochs. However, the dynamics of neural activity vary at a finer timescale, and we wished to examine how the representation of task conditions may vary in neural responses over time. We evaluated the effective temporal coding dimensionality of each neuron using principal component analysis (PCA) (Methods). We then determined the effective dimensionality of neural activity, *N*_eff_, as a measure that penalizes small eigenvalues that could arise because of noise. Higher *N*_eff_ values suggest a high coding dimensionality^37^. A cell with a high temporal dimensionality can have a time-varying magnitude of stimulus selectivity or changes in its order of stimulus selectivity over time (for example, mixed selectivity). Effective dimensionality during encoding of visual stimuli increased before it plateaued, showing a significantly higher temporal coding dimensionality in mature PFC neurons (*F*_(1, 354)_ = 3.66, *P* = 0.000156 (GAMM); significant developmental improvements −31 to 8 months relative to the mid-adolescence age (Fig. 3a)).

In recent years, it has been recognized that population measures of neural activity contain information about stimuli and tasks that may not be apparent in single-neuron measures. Higher dimensionality of population responses, in particular, has been linked to greater cognitive flexibility^38,39^, which is known to improve in adolescence^40^. Thus, we hypothesized that neural activity followed a high dimensional trajectory not only in single-neuron temporal space, but also within the full neural state space. Therefore, we calculated the dimensionality of neural responses in the full *N*-dimensional neural space (where *N* is the number of neurons in each maturation interval; Methods) across different maturation intervals. Indeed, the dimensionality of the full neural space increased over adolescent maturation (*F*_(1, 9999)_ = 8,079, *P* < × 10^−16^ (GAMM); significant increase −31 to −1 months relative to mid-adolescence age with peak at −1 month (Fig. 3b)).

The PFC maintains working memory information and manages cognitive processes, such as suppressing irrelevant stimuli or distractors during task performance, by dynamically representing stimuli in a task-specific manner^41,42^. The same stimuli can be represented in different subspaces when used in the context of different tasks, for example, creating distinct dimensions for sensory and memory representations to mitigate interference between sensory inputs and memory representations^39,43^. As working memory and resistance to distractors becomes increasingly efficient during adolescent development, we speculated that the representation of cue and distractor stimuli may be rotated orthogonally in the neural population space; this relative rotation may improve with maturation, endowing the PFC with the ability to better filter out distractors. Therefore, we used the distractor task (Fig. 1b), which required animals to view stimuli drawn from an identical set of locations and to keep track of the context of the sensory inputs. In each maturation interval, we applied PCA on a firing rate matrix constructed with each neuron’s average firing rate to each stimulus separately in cue and distractor epochs. The first three principal components generally explained more than 50% of the variance. To ensure fair comparisons between different populations of neurons at each maturation interval, we reduced the population firing pattern to the first three dimensions to measure the representation structure in low-dimensional space. We quantified the differences in representation of the same stimuli based on the angle between subspaces defined by the different task context (target or distractor; Fig. 3c and Methods). The acute angle between the target and distractor subspaces is the largest around mid-adolescence age (peaking at −4 months relative to the mid-adolescence age). The rotation between the planes defined by the stimuli when they appeared during the cue and distractor periods increased in early adolescence and decreased slightly before plateauing with a significant angle of rotation (*F*_(1, 7499)_ = 343.5, *P* < 2 × 10^−16^ (GAMM); significant increase −30 to −4 months relative to the mid-adolescence age (Fig. 3d)).

### Structural brain changes predict adolescent cognitive improvements

Two types of structural brain changes uncovered by imaging methodologies have been linked to improvements in neural activity and computation. Pruning of infrequently used synapses^12^, which can produce a progressive decrease in cortical volume and thickness^8^, improves the efficiency and stability of neural activity associated with cognitive functions^44,45^. Similarly, myelination is modulated in an activity-dependent fashion^46^ and can enhance the maintenance of working memory-related activity^47^. Therefore, we wished to determine the aspects of brain structural changes that best explain the changes in neural activity and behavior we observed in adolescence. As neurophysiological recordings were obtained from these animals, we performed volume measurements of cortical and subcortical structures on the hemisphere opposite to the one where recordings were performed.

**Fig. 4:**
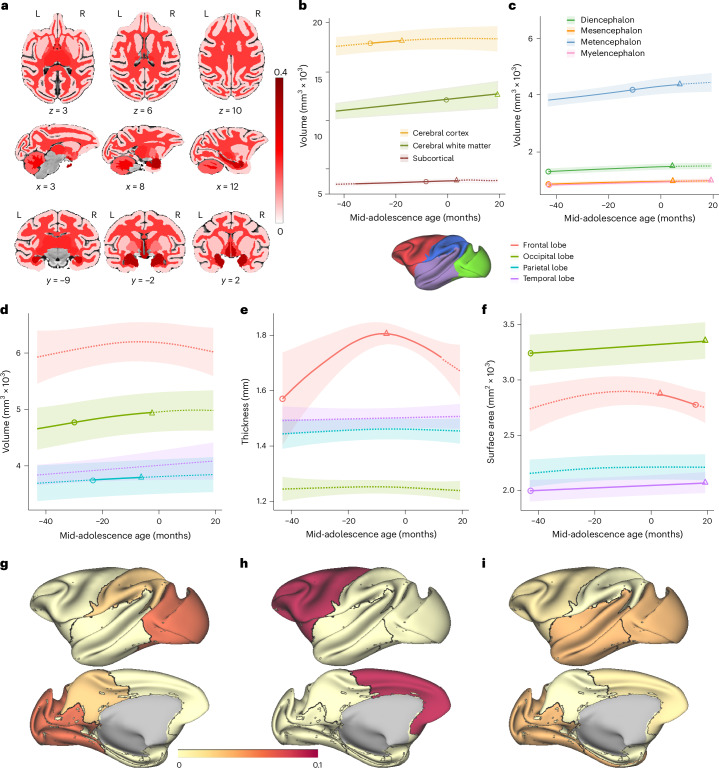
Longitudinal developmental trajectories of the macaque brain. **a**, Map of volume changes for white and gray matter and subcortical areas. **b**,**c**, The dashed curves represent volumes of brain segmentations (**b**) and structures (**c**) as a function of age. **d**–**f**, In each lobe, segmented using level 1 of the Cortical Hierarchy Atlas of the Rhesus Macaque (CHARM), the dashed curves represent volume (**d**), thickness (**e**) and surface area (**f**) as a function of age. The solid curves indicate the significant developmental effect of the GAMM fittings. The shaded ribbons denote the 95% CIs. The circle denotes the time of peak development velocity. The triangle denotes the time of maximum value. **g**–**i**, Proportional change during adolescence compared with different lobes for volume (**g**), cortical thickness (**h**) and surface area (**i**). The color represents the proportional change of each lobe relative to the earliest data points (−43 months to the mid-adolescence age). Source data

**Fig. 5:**
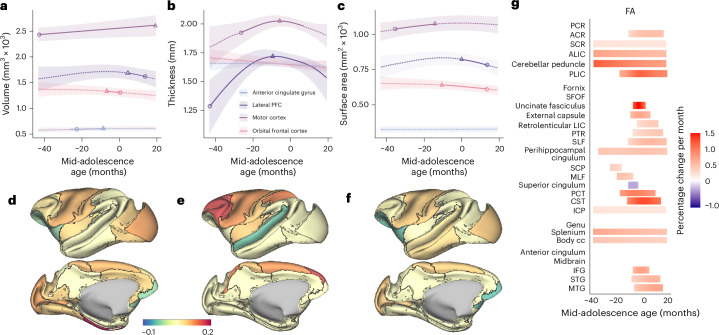
Structural changes during development in the frontal lobe and white matter tracts. **a**–**c**, The dashed curves represent the longitudinal developmental trajectories of areas in the frontal lobe for volume (**a**), cortical thickness (**b**) and surface area (**c**). The solid curves represent the fit of mean values and indicate the significant developmental effects of the GAMM fittings. The shaded ribbons denote the 95% CIs. The circle denotes the time of peak development velocity. The triangle denotes the time of maximum volume. **d**–**f**, Proportional change during adolescence compared with different brain areas in level 2 of the CHARM atlas for volume (**d**), cortical thickness (**e**) and surface area (**f**). The color represents the proportional change of each lobe relative to the earliest data points (−43 months to the mid-adolescence age). **g**, Stages of significant change in FA and timing of maturation for major white matter tracts. Each row is a region of interest (ROI) grouped to projection, association, commissural tracts, brain stem white matter regions and short-range white matter. Rows are sorted according to the time of maturation in each group, which is defined as the time in which the rate of change was no longer significantly different than zero. Colors represent the percentage change per month (red = increase, blue = decrease). List of abbreviations for white matter tracts in **g** can be found in Supplementary Table 10. Source data

**Extended Data Fig. 3:**
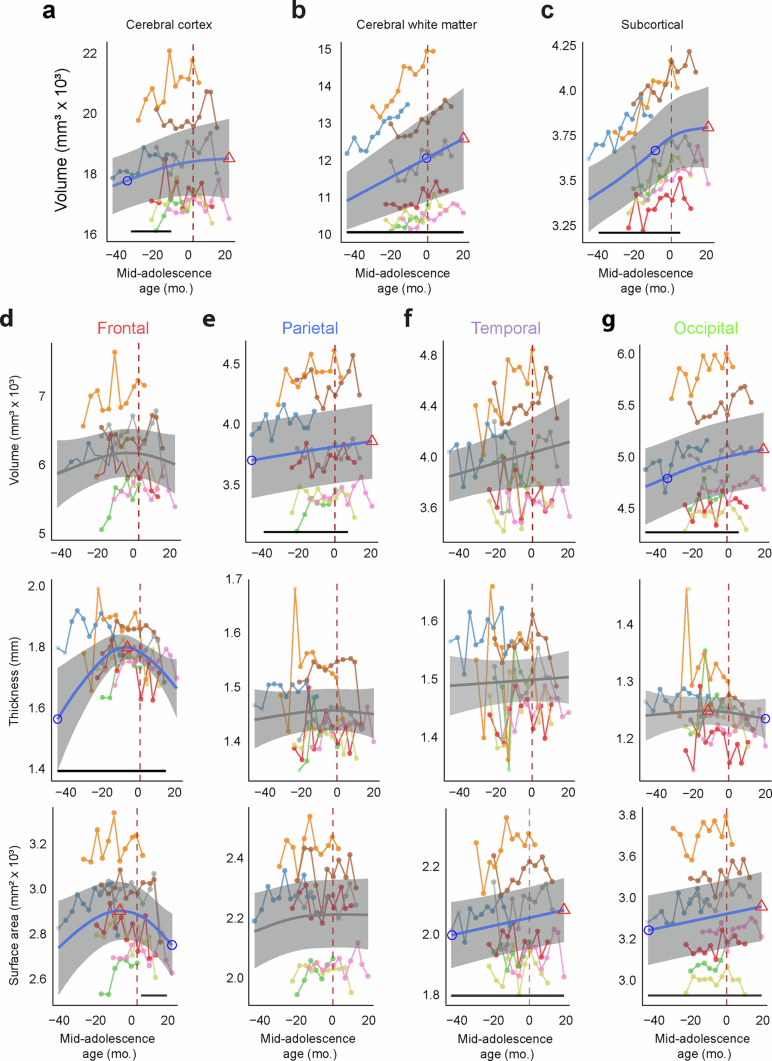
Raw data and fitted structural developmental trajectories of brain segmentations and lobes. (**a**) Total gray matter (cerebral cortical) volume as a function of age. (**b**) As in a, for white matter volume as a function of age. (**c**) As in a, for subcortical volume as a function of age. (**d**-**g**)Volume, thickness and surface area of the four lobes of the cerebral cortex as a function of age. In each panel, top: cortical volume; middle: cortical thickness; bottom: surface area. ROIs were determined using Charm atlas level 1. Blue or gray curve indicates the GAMM fitted trajectory. Gray shaded regions denote the 95% confidence intervals (CIs). Blue circle denotes the time of peak development velocity. Red triangle denotes the time of maximum value. Dashed vertical line denotes the mid-adolescence age 0. The horizontal bar denotes significant developmental effect intervals. Source data

**Extended Data Fig. 4:**
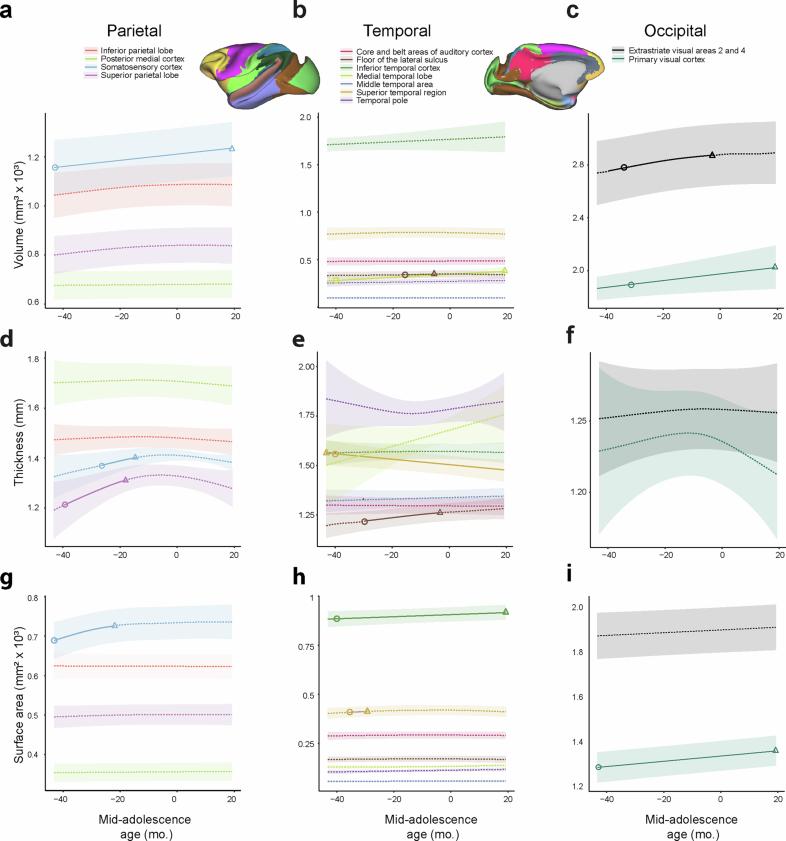
Structural changes of areas during development in parietal, temporal and occipital lobes. (**a**, **d**, **g**) Cortical volume (**a**), thickness (**d**) and surface area (**g**) of areas in parietal lobe as a function of age. (**b**, **e**, **h**) Cortical volume (**b**), thickness (**e**) and surface area (**h**) of areas in temporal lobe as a function of age. (**c**, **f**, **i**) Cortical volume (**c**), thickness (**f**) and surface area (**i**) of areas in occipital lobe as a function of age. In each plot, curve indicates the GAMM fitted trajectory of one area in Charm 2 atlas in each lobe. Solid curves indicate significant developmental effect of the GAMM fittings. Shaded ribbons denote the 95% confidence intervals (CIs). Circle denotes the time of peak development velocity. Triangle denotes the time of maximum volume. Source data

**Extended Data Fig. 5:**
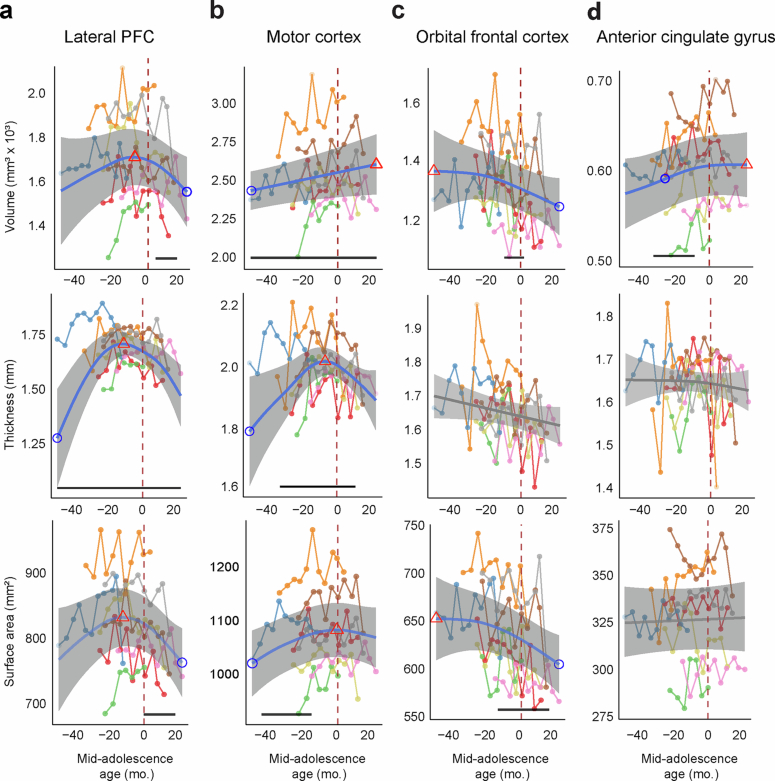
Raw data and fitted structural developmental trajectories of areas in frontal lobe. (**a**-**d**) Volume, thickness and surface area of the areas in frontal lobe as a function of age. In each panel, top: cortical volume; middle: cortical thickness; bottom: surface area. ROIs were determined using Charm atlas level 2. In each plot, blue or gray curve indicates the GAMM fitted trajectory. Gray shaded regions denote the 95% confidence intervals (CIs). Blue circle denotes the time of peak development velocity. Red triangle denotes the time of maximum value. Dashed vertical line denotes the mid-adolescence age 0. The horizontal bar denotes significant developmental effect intervals. Source data

**Extended Data Fig. 6:**
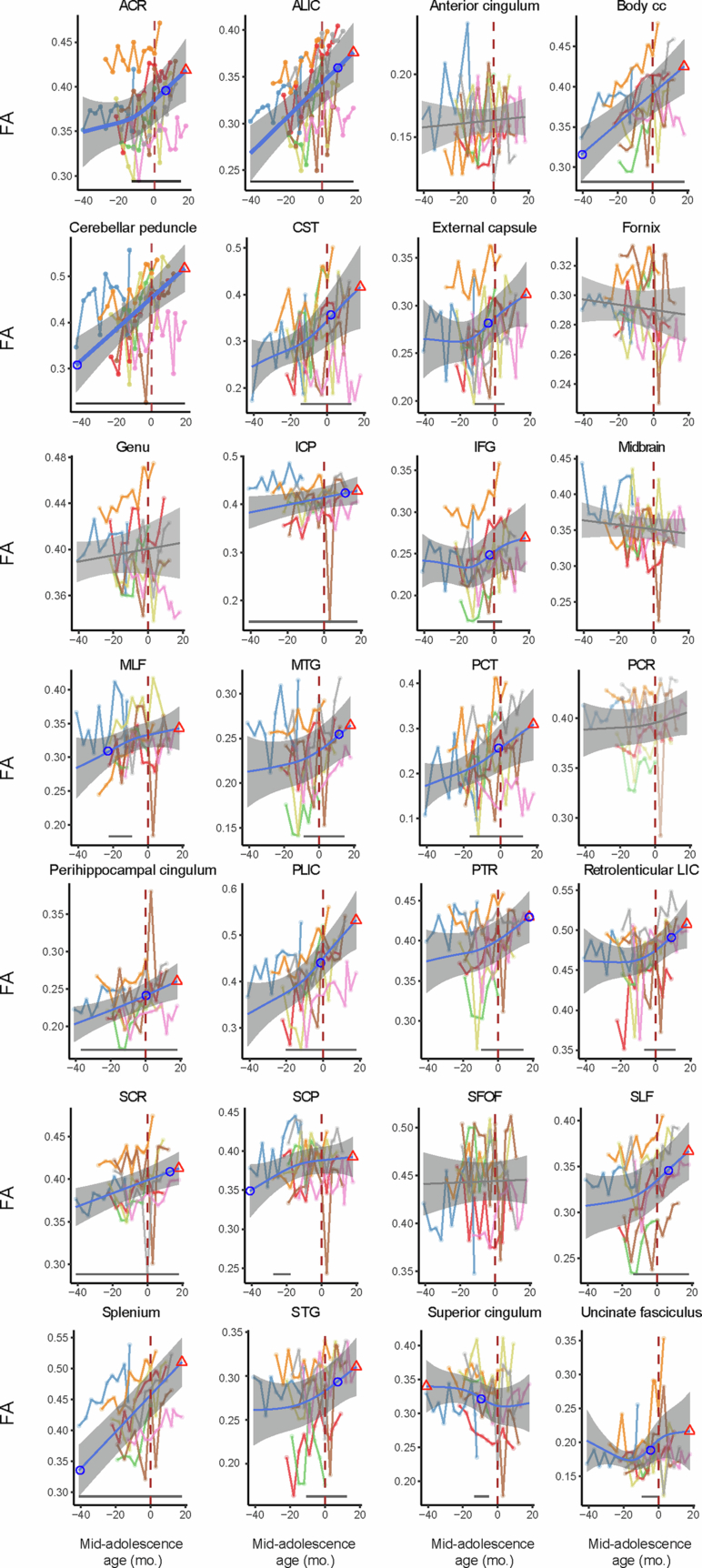
Raw data and fitted FA developmental trajectories. Each dot represents the Fractional Anisotropy value from one subject in one session. In all plots, blue or gray curve indicates the GAMM fitted developmental trajectory. Gray shaded regions denote the 95% confidence intervals (CIs). Blue circle denotes the time of peak development velocity. Red triangle denotes the time of maximum value. Dashed vertical line denotes the mid-adolescence age 0. The horizontal bar denotes significant developmental effect intervals. Panels were sorted in alphabetical order. Source data

**Extended Data Fig. 7:**
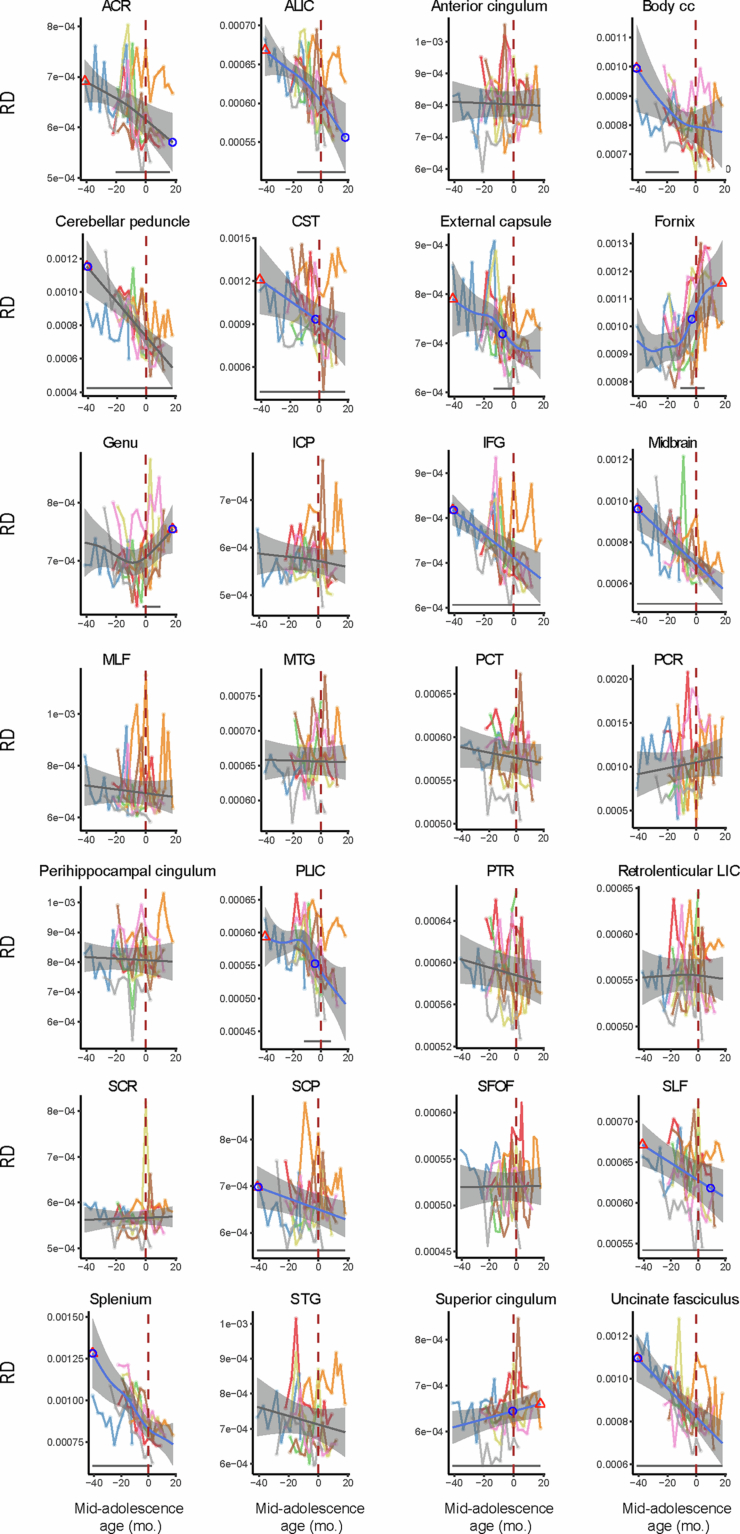
Raw data and fitted RD developmental trajectories. Each dot represents the Radial Diffusivity value from one subject in one session. In all plots, blue or gray curve indicates the GAMM fitted trajectory. Gray shaded regions denote the 95% confidence intervals (CIs). Blue circle denotes the time of peak development velocity. Red triangle denotes the time of maximum value. Dashed vertical line denotes the mid-adolescence age 0. The horizontal bar denotes significant developmental effect intervals. Panels were sorted in alphabetical order. Source data

**Extended Data Fig. 8:**
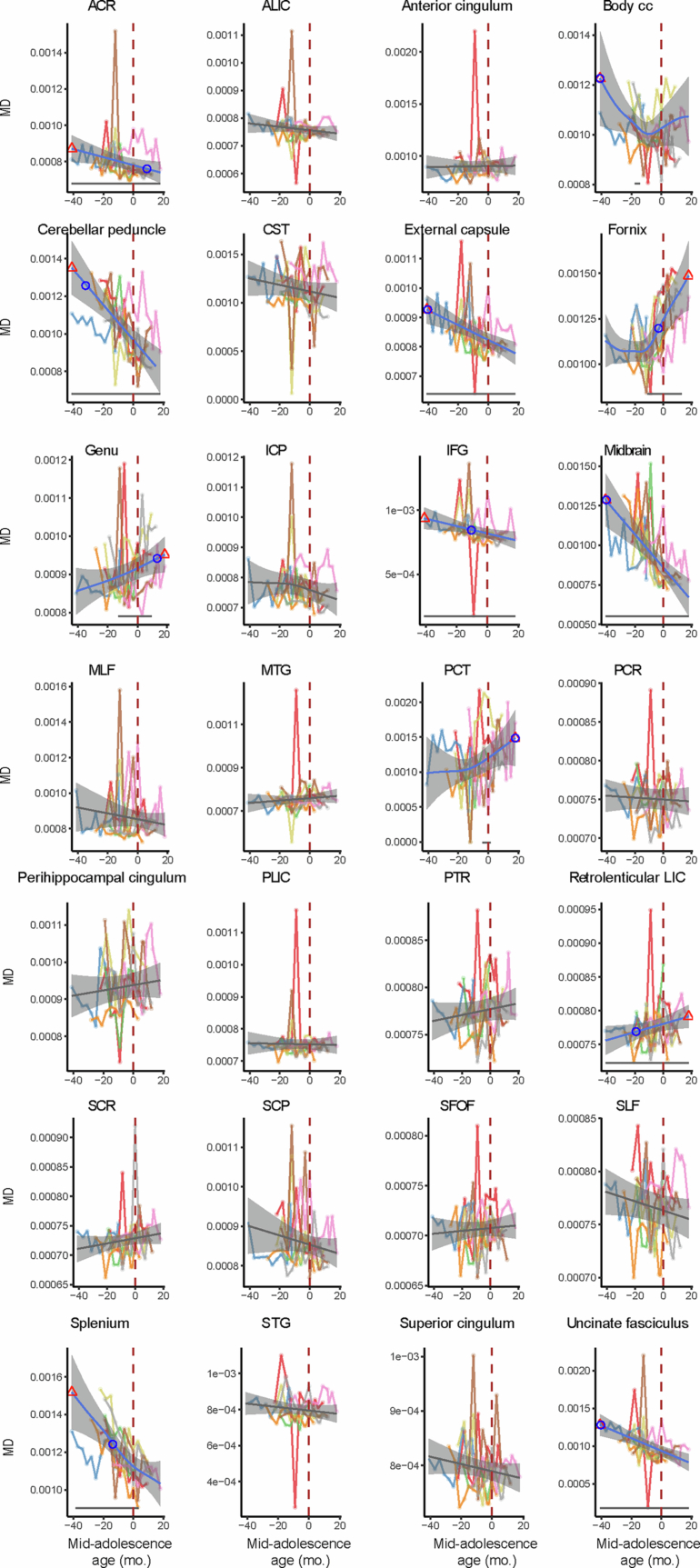
Raw data and fitted MD developmental trajectories. Each dot represents the Mean Diffusivity value from one subject in one session. In all plots, blue or gray curve indicates the GAMM fitted trajectory. Gray shaded regions denote the 95% confidence intervals (CIs). Blue circle denotes the time of peak development velocity. Red triangle denotes the time of maximum value. Dashed vertical line denotes the mid-adolescence age 0. The horizontal bar denotes significant developmental effect intervals. Panels were sorted in alphabetical order. Source data

We first considered global brain measures for white and gray matter (Extended Data Figs. 3–5 and Supplementary Figs. 4–7), subcortical areas (Supplementary Figs. 8 and 9) and fiber tracts (Extended Data Figs. 6–8). Overall, we observed increases in all brain structure volumes (Fig. 4a–c). We also examined more specific measures in brain areas implicated with working memory, including the lateral PFC (Fig. 5). In humans, gray matter (cortical) volume peaks during childhood and decreases thereafter^8^, thought to be driven by processes such as synaptic pruning. In monkeys, a global volume maximum is also observed in childhood (0.74 years); however, many areas exhibit a second period of increase in adolescence^9^. Indeed, in our cohort, which was tracked after year 3, we observed increases in all brain structure volumes of the macaque’s brain (Fig. 4a–c). We found that whole-brain cortical gray matter volume increased in our dataset (*F*_(1, 75)_ = 4.2, *P* = 0.0467 (GAMM); significant increase −33 to −12 months relative to the mid-adolescence age (Fig. 4b)). Peak gray matter volume was observed at a chronological age of 78 months, in late adolescence. Although the volume increase was relatively small in the cerebral cortex (3.5% from −43 to 20 months relative to the mid-adolescence age (Fig. 4b)), it was more pronounced in non-cortical areas and particularly white matter (15.3%) (Fig. 4b), subcortical structures (10.0%) (Fig. 4b), diencephalon (20.0%) (Fig. 4c), mesencephalon (22.0%) (Fig. 4c), myelencephalon (39.0%) (Fig. 4c), cerebellum (12.5%) (Supplementary Fig. 7b) and cerebellum white matter (21.6%) (Supplementary Fig. 7c). Cerebral white matter exhibited a relatively linear increase in adolescent monkeys (*F*_(1, 75)_ = 25.8, *P* = 2.5 × 10^−6^ (GAMM); significant increase −43 to 20 months relative to the mid-adolescence age (Fig. 4a,b)), as observed in humans.

As expected, the variation in lobar cortical volume (Fig. 4g), thickness (Fig. 4h) and surface area (Fig. 4i) was minimal, corresponding to the relatively small variation in whole-brain cortical volumes. However, the variation and the inverse U-shape of the cortical thickness of the frontal lobe stands out when compared to the metrics or other lobes (Fig. 4e). To investigate this further, we focused on the finer segmentation of brain regions and fiber tracts associated with working memory and the frontal cortex (Fig. 5a–f, Extended Data Fig. 5 and Methods). The thickness of the lateral PFC and motor cortex increased early on and peaked before the mid-adolescence age, followed by a decrease later in adolescence (Fig. 5b,e). This was also the case in the posterior parietal cortex, a region functionally and anatomically highly interconnected with the lateral PFC (Extended Data Fig. 4d and Supplementary Fig. 4). In contrast, most of the other frontal cortical regions, including the orbitofrontal cortex and anterior cingulate cortex, did not show any significant change in cortical thickness with maturation (Fig. 5b,e). Most regions in the other lobes (parietal, temporal, occipital) did not show significant variations in the three metrics, except for primary areas, such as the somatosensory cortex (Extended Data Fig. 4 and Supplementary Fig. 4) and the primary visual cortex (Extended Data Fig. 4 and Supplementary Fig. 6), as well as the previously mentioned motor cortex (Fig. 5b,e and Extended Data Fig. 5b).

Even for the lateral PFC, the volume of which exhibited an overall decrease, volume did not predict behavioral performance well (DI versus volume: *r* = 0.132, permutation test, *P* = 0.394; RT versus volume: *r* = −0.171, *P* = 0.1800). Thickness and surface area were only weakly correlated with measures of behavioral performance (DI versus thickness: *r* = 0.031, *P* = 0.963; RT versus thickness: *r* = −0.299, *P* = 0.008; DI versus surface: *r* = 0.422, *P* < 0.0001; RT versus surface: *r* = 0.106, *P* = 0.569).

**Fig. 6:**
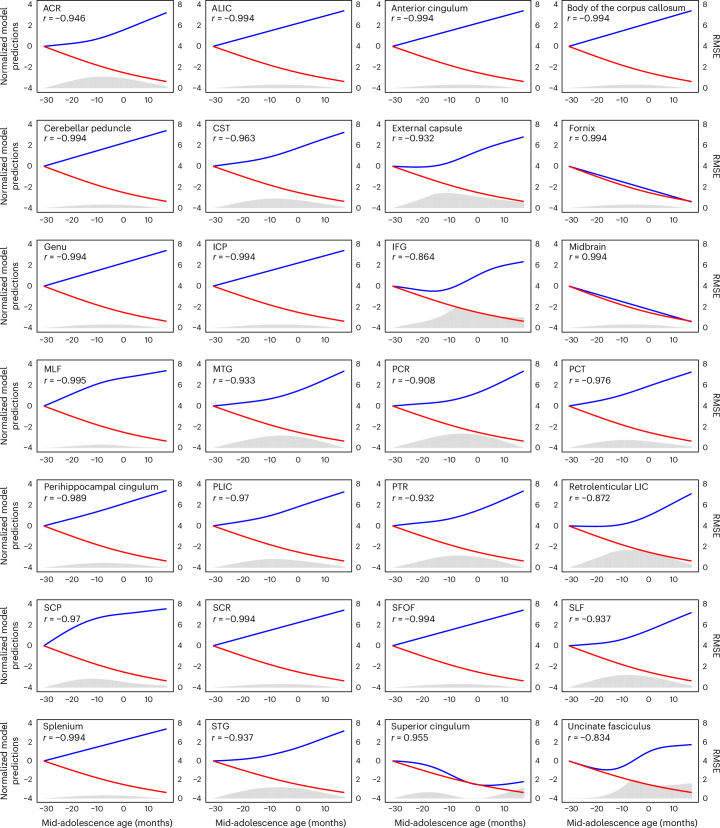
Correlation between the GAMM-fitted trajectories of the DI and FA of each white matter tract. The blue curve represents the normalized GAMM-fitted trajectory of the FA of the tract; the red curve represents the normalized GAMM-fitted trajectory of the DI in the ODR task. The histogram indicates the root mean square error (RMSE) between corresponding prediction values of the GAMMs. Source data

**Extended Data Fig. 9:**
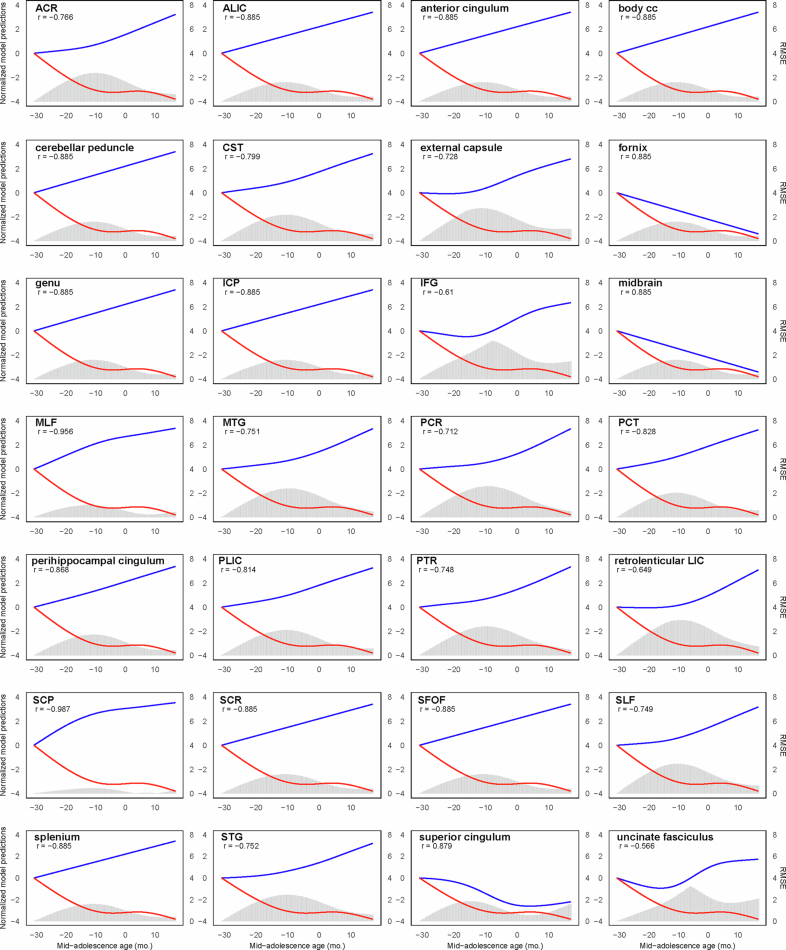
Correlation between the GAMM fitted trajectories of RT and FA of each white matter tract. In each panel, blue curve represents normalized GAMM fitted trajectory of the FA of the tract and red curve represents normalized GAMM fitted trajectory of RT in ORD task. Histogram indicates RMSE between corresponding prediction values of the GAMMs. Source data

**Extended Data Fig. 10:**
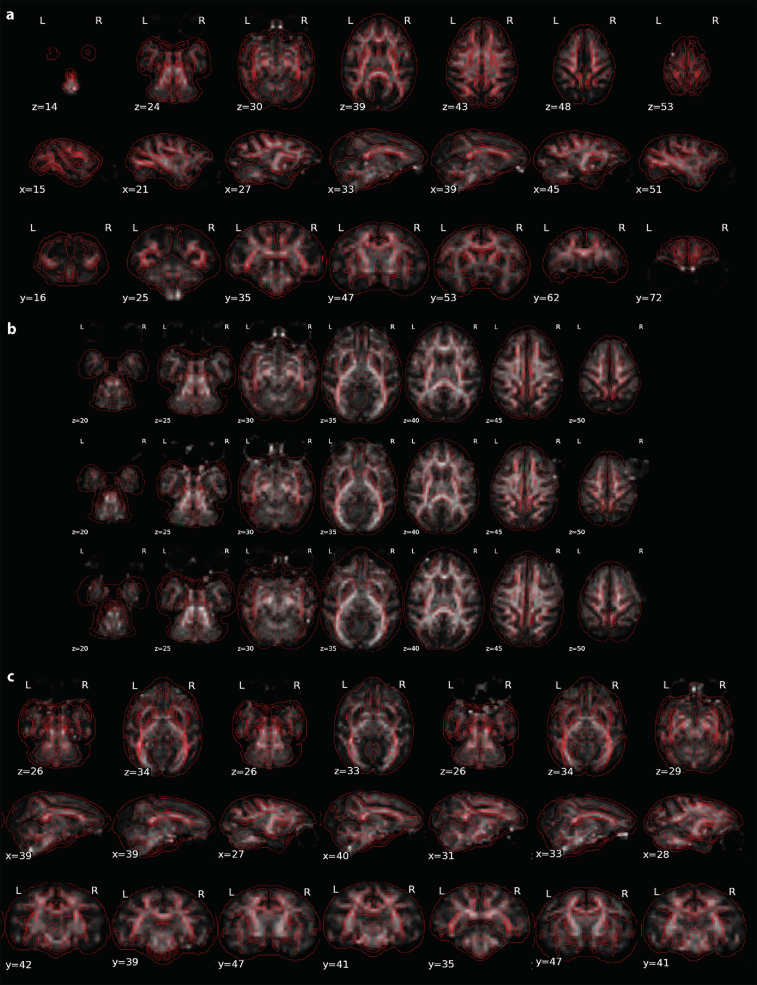
Tract registration. The grayscale underlay represents the FA image and the red contour is outlining the DTI template^84^. FA images were registered to the T1-weighted image and then aligned to the DTI template. (**a**) Registration data for a single monkey (subject U) in axial, sagittal, and coronal views. (**b**) Longitudinal registration for subject O, showing alignment at three time points, each one year apart. (**c**) Registration across multiple subjects (O, P, Q, R, S, T, V), displayed in comparable slices from left to right, respectively.

Developmental effects on white matter maturation, quantified using fractional anisotropy (FA), radial diffusivity (RD) and mean diffusivity (MD), were evident across the brain, with most tracts reflecting increases in FA and decreases in RD and MD with development (Fig. 5g and Supplementary Fig. 10). We also examined in more detail the tract maturation that most closely paralleled the trajectory of working memory performance improvement and prefrontal cortical activity changes. We performed correlation analyses between the tracts we identified and the behavioral performance of the ODR task, including DI and RT. FA changes in several white matter tracts had a high negative correlation (*r* > 0.9) with the precision (DI) of behavioral performance in the ODR task (Fig. 6). These tracts include the middle longitudinal fasciculus, the superior fronto-occipital fasciculus and the anterior cingulum, which projected from or to the frontal lobe (permutation test: *P* < 0.0001 in each case). Maturation of several other tracts had similar trajectories and showed correlation with behavioral performance (anterior limb of the internal capsule and superior corona radiata, cerebellar peduncles, and genu and splenium of the corpus callosum). Strong negative correlations were also observed between FA trajectories with RT during adolescence (Extended Data Fig. 9; tract registrations are shown in Extended Data Fig. 10).

To further assess how these measures jointly predict task performance, we used a mixed-effects random forest (MERF) and used SHapley Additive exPlanations (SHAP) values for interpretability. Consistent with our correlation findings, MERF revealed that the PFC baseline activity and FA values of the identified tracts had a prominent role in explaining both DI and RT outcomes, with most FA values having a higher prediction power than the volumes of brain regions (Supplementary Tables 8 and 9). This multivariate approach confirms and extends our correlation results by demonstrating that developmental changes in specific white matter tracts, together with other brain structural measures, robustly predict cognitive performance improvements across adolescence.

## Discussion

Human cognitive ability improves substantially from childhood through adolescence^4,36^, but until now the underlying changes in neural activity have not been well investigated. In this study, we shed light on the processes underlying cognitive maturation, using nonhuman primates as a model. Monkeys, like humans, exhibited substantial variability in development. Therefore, we relied on skeletal assessment to best physical development and align the growth trajectories of different individuals. This alignment also allowed us to account for systematic sex differences in developmental trajectories. We used an ostensibly simple task to track the trajectory of working memory function, using the precision and latency of responses in the ODR task as the primary behavioral outcomes. This choice allowed us to track performance from the earliest time points, as all animals were able to master this readily and to compare results with the extensive human childhood and adolescence literature^4,5^. Critically, recent studies showed that these variables track adolescent development remarkably well across a range of abilities, including much more complex processes such as planning, response inhibition and other aspects of executive function^7^. Neuropsychiatric conditions and mental illnesses that emerge in adolescence to early adulthood, most notably schizophrenia, are also associated with decreased performance in working memory tasks^48^. Our results revealed a progressive improvement in both precision and latency (defeating a speed–accuracy trade-off) with a shape that closely resembled human development.

Neurophysiological recordings from the PFC revealed changes in neural activity that could account for these behavioral changes. Our current results suggest that changes even in the baseline firing rate of prefrontal neurons during the fixation period of the task could capture well the trajectory of behavioral improvement. However, strong evidence exists that the firing rate in the delay period is the strongest determinant of performance of working memory tasks^49^; prior studies demonstrated increased activity in the delay period of working memory tasks, even above the baseline^23^. Computational models suggest that recurrent connections between prefrontal neurons allow activity to reverberate in the network during working memory tasks, and increased synaptic drive will generally allow the network to enter a multi-stability regime more readily^50,51^. The improvement in saccade precision metrics indicates that decrease in trial–trial variability is an important developmental milestone. Indeed, fMRI measures related to fluctuations in the amplitude of task-related brain states stabilize during development^52^. Excessive variability is also a sensitive marker of cognitive function in conditions such as schizophrenia^53^ and attention-deficit/hyperactivity disorder^54^. We now tie these behavioral variability measures to changes in neural variability metrics, including the CV of ISIs and Fano factor. Reduced behavioral and firing rate variability can also be mapped to a stronger synaptic drive in neural networks, which renders the peak of activation in the network less susceptible to drift in each trial^55^. Variability may also reflect a period of plasticity in adolescence as circuits are engaged differently to identify optimal function into adulthood in a Hebbian process selecting circuitry that is most highly used because of its efficacy.

Our analysis also revealed more complex measures of neural activity that change during adolescent development. The dimensionality of neural representations increased, as did the extent of rotation of stimulus representations when these were presented as cue stimuli, or as distractors, in a variant of the ODR task. Higher dimensionality of population responses, and the related phenomenon of nonlinear mixed selectivity, have been linked to greater cognitive flexibility^38,56^. Rotation of stimulus representation is a critical mechanism in suppressing irrelevant stimuli or distractors during task performance by dynamically representing stimuli in a task-specific manner^39,41–43^. Such changes observed during development now account for cognitive improvements in domains such as cognitive flexibility and ability to withstand distraction^22^, which improve through adolescence.

Recent structural imaging studies have codified the trajectory of age-related brain changes across cohorts of hundreds of individual humans^8^ and monkeys^9^. A hallmark of brain development is the decrease of cortical volume and thickness after childhood^10^, thought to be driven by pruning of infrequently used synapses^12^, although the apparent decrease in thickness may also be driven by myelination of gray matter fibers^57^. Pruning confers clear improvements in neural computations, including stronger stability and improved efficiency of neural representations^44,45^; it is probably a factor during childhood development. However, decreases in gray matter volume were not drivers of the adolescent working memory enhancement that we observed.

Instead, our findings pointed out an association between the maturation of white matter tracts and improvements in working memory performance, as well as prefrontal cortical activity during adolescence. These results align with the existing literature highlighting the prolonged maturation of white matter pathways in humans, which continues well into early adulthood^58,59^.

The FA increases we observed in tracts connecting the frontal lobe to cortical and subcortical regions are consistent with findings that show an association between the maturation of white matter and improvements in cognitive tasks that depend on these connections, such as working memory and executive functions. At the cellular level, diffusion tensor imaging (DTI) measures, including FA and MD, are correlated with myelination levels; the postnatal development of myelination parallels closely that of DTI measures^60^. Postnatal myelination is plastic and modulated in an activity-dependent fashion^46,61^. Myelination-dependent increase of conduction velocity^62^ and spike arrival time synchronization^63^ could support cognitive functions dependent on the processing of top-down and bottom-up information by the PFC via cross-regional neuronal synchronizations at characteristic frequencies^64,65^. The importance of intercortical connectivity in the case of working memory has been further demonstrated via multiregional modeling, highlighting its role in sustaining persistent activity when local recurrent connectivity is weak and when the system faces distractions^47,66^. Other factors implicated in distractor-resistant working memory including spine count and dopamine D1 receptor distribution; these are also likely to develop over this period^47,67,68^.

Some limitations apply to our conclusions. Although nonhuman primates provide the closest animal models to humans, there may still be differences in the rate of cognitive development; humans also have much greater capacity for cognitive functions. Systematic structural changes are also present between species, including in the trajectory of gray matter volume across development^8,9^. Although our findings indicate extensive structural maturation during adolescence, direct correlations between these structural changes and improvements in cognitive functions were limited. This apparent discrepancy may stem from the complex, multifactorial nature of cognitive function development, which probably involves not only macroscopic anatomical maturation but also functional connectivity changes, synaptic plasticity and refinements in neural circuitry that are not fully captured by volumetric or cortical thickness measures alone. Indeed, our analyses revealed that white matter maturation, quantified through measures like FA, showed a stronger and more consistent association with cognitive improvements than gray matter structural changes. Future studies integrating high-resolution structural imaging with functional connectivity analyses and finer-grained neural recordings might provide a more complete understanding of how structural brain development directly contributes to cognitive enhancement during adolescence. Finally, our study was not sufficiently powered to detect potential sex differences in development; future studies with longer scan times can reveal anatomical changes in more detail.

Nonetheless, our results provide new evidence for substantial neural maturation through adolescence into adulthood and the mechanisms that underlie them. Disruptions in the maturation of these systems may have an important role in psychopathology that emerges at this time (for example, schizophrenia, mood disorders, substance use disorders) and are typically associated with impairments in executive function.

## Methods

### Animals

Behavioral, imaging and neurophysiological recordings were obtained from 14 (ten male and four female) rhesus monkeys (*M. mulatta*). We distinguished between three cohorts: cohort A included eight monkeys (six males, two females) and was tracked throughout adolescence. Cohort B included four monkeys (four males) and was tested at two time points (early adolescence, adulthood), providing a control for training exposure. Cohort C included two female monkeys that were first trained in adulthood and provided a control for age without training. No statistical methods were used to predetermine sample sizes but our sample sizes are similar to those reported in previous publications^23,24^. As the developmental stage of animals was evident, data collection and analysis were not performed blind to the conditions of the experiments. All surgical and animal use procedures were reviewed and approved by the Institutional Animal Care and Use Committees of Wake Forest University and Vanderbilt University, in accordance with the U.S. Public Health Service Policy on Humane Care and Use of Laboratory Animals and the National Research Council’s Guide for the Care and Use of Laboratory Animals.

### Developmental profiles

We tracked the developmental measures of cohort A monkeys on a quarterly basis before, during and after neurophysiological recordings. Monkeys of this cohort were first obtained at an age of 2.3–2.9 years from a commercial breeding company (Alpha Genesis) where they were mother-reared and lived in species-typical social groups. Once in the laboratory, they were housed in groups of 2–3 animals, in view of each other, and other conspecifics. Like previous studies^23,24^, we obtained morphometric measures, including body weight, trunk length, femur length, canine eruptions and length. Testicle length, width and volume was additionally determined for male monkeys (with a Prader orchidometer, ESP Limited); nipple length was determined for female monkeys. To determine each monkey’s developmental progress, we determined bone maturation using X-rays of the upper and lower extremities, and assayed hair concentration of hormones, including testosterone and dihydrotestosterone. We relied primarily on skeletal assessment to best capture physical development^35,69,70^ and align the growth trajectories of different individuals. Using these measures, we defined a mid-adolescence age for each monkey, defined as the time of each monkey’s distal tibial epiphyseal closure, as observed by veterinary professionals evaluating the X-rays, blinded to findings of other aspects of the study.

### Behavioral tasks

Monkeys were trained to perform an ODR task^49^. This is a spatial working memory task that requires the animal to remember the location of a cue stimulus appearing on a screen for 0.5 s (Fig. 1a). The cue stimulus was a 1° white square appearing at one of eight locations arranged on a circle of 10° eccentricity, spaced by 45 degrees (Fig. 1c). After a 1.5-s delay, the fixation point was extinguished, indicating the monkey to make a saccade to the remembered location of the cue within 0.6 s. The saccade needed to terminate on a 6° radius window centered on the stimulus; the monkey was required to hold fixation within this window for at least 0.1 s. Animals were rewarded with liquid rewards (typically fruit juice) for successful completion of a trial.

Eye position was monitored with an infrared eye tracking system (sampling rate = 240 Hz, RK-716, ISCAN). Breaking fixation at any point before the offset of the fixation point aborted the trial and resulted in no reward. The visual stimulus display, monitoring of eye position and synchronization of stimuli with neurophysiological data were performed using in-house software and were implemented with MATLAB^71^.

Four of the eight monkeys of cohort A (one female and three males) were additionally trained on the ODR task with a longer delay of 3.0 s after they were capable of performing the shorter task. The same four monkeys were also trained to perform a variant of the task, the ODR with distractor task^72^. This task involved a distractor, presented 1.25 s after the cue for 0.5 s, followed by another 1.25-s delay before the fixation point was off, making the total duration of the trial the same as in the ODR task with the 3-s delay. The cue could appear at one of four locations arranged on a circle of 10° eccentricity, spaced by 90°. For each cue location, five distractor conditions were used: the location of the distractor could appear at a location diametric to the cue (180°), 90° counterclockwise to the cue (90°), 45° counterclockwise to the cue (45°) or the same as the cue (0°). The fifth condition involved no distractor presentation.

### Recording phases

The monkeys were initially trained in the tasks mentioned above before their neurophysiological recordings. They were naive to behavioral training or task execution of any kind before the behavioral training. Once the animals had reached asymptotic performance, behavioral and neurophysiological recordings were obtained from the monkeys in cohort A at time points spaced approximately 3 months apart, from 3.4 to 6.2 years old. Each behavioral time point of each animal contained an average of 19 sessions. Between time points, animals were returned to their colony and were not tested or trained in any task until their next time point.

### Surgery and neurophysiology

After the animals of cohort A had reached asymptotic performance in the behavioral tasks for the first time, we implanted a 20-mm diameter recording cylinder over the PFC of each monkey. Localization of the recording cylinder was based on MRI, processed with the Brainsight system (Rogue Research). Recordings at each time point (see the ‘Recording phases’ section) were collected with glass or epoxylite-coated Tungsten electrodes with a diameter of 250 μm and an impedance of 4 MΩ at 1 kHz (FHC). Electrode penetrations within the cylinder were placed with a stereotaxic grid system (Crist Instruments) to ensure we precisely and evenly sampled from the ROI at each time point, without visiting the exact same grid location to avoid accumulated damage. Within this grid, neurons were sampled in an unbiased fashion, recording from all neurons encountered in our penetrations, without an effort to select some neurons based on any functional properties. Electrical signals recorded from the brain were amplified, band-pass-filtered between 500 Hz and 8 kHz, and stored through a modular data acquisition system at 25-μs resolution (APM system, FHC).

Recordings were obtained and analyzed from areas 8a and 46 of the dorsolateral PFCs. Recorded spike waveforms were sorted into separate units using a semiautomated cluster analysis method based on the KlustaKwik algorithm^73^. Neurons for which at least four correct trials in every stimulus condition were available in the ODR task were used in the following analyses. Data distribution was assumed to be normal but this was not formally tested. Other than the minimum trial requirement, no data from any particular animals or data points were excluded from the analysis.

### MRI acquisition and processing

Structural MRIs were collected from the monkeys of cohort A every 3 months from 2.8 years (34 months) of age to 5.8 years (69 months) of age. In preparation for the MRI scan, anesthesia was administered using ketamine (5–10 mg kg^−1^) and dexmedetomidine (0.015 mg kg^−1^), and was maintained using isoflurane. Animals were intubated and artificially ventilated at about 20 breaths per minute. Expired CO_2_ was monitored and maintained between 35 and 45 mmHg. Animals were scanned under isoflurane anesthesia at 1–1.5%. Heart rate and oxygen saturation levels were monitored using a pulse oximeter. Body temperature was maintained using warm blankets. The MRI system was a 3 Tesla Siemens MAGNETOM Skyra (Siemens Healthcare). Anatomical images were acquired using a T1-weighted magnetization-prepared rapid gradient-echo sequence: repetition time (TR) = 2,700 ms, echo time (TE) = 3.32 ms, inversion time = 880, field of view (FOV) = 128 × 128 mm, 192 slices of 0.5-mm thickness, resolution = 0.5 mm isotropic. Resting state time series data were also acquired using a multiband echo-planar imaging sequence: TR = 700 ms, TE = 32.0 ms, flip angle = 52°, repetitions = 700, FOV = 128 × 128 mm, 32 slices, resolution = 2 mm isotropic.

Spatial preprocessing was performed using the EvoDevo NeuroImaging Explorer (EDNiX), a new pipeline coded in Python, which relied on functions from Analysis of Functional Neuroimages (AFNI), Advanced Normalization Tools^74^, FMRIB Software Library^75^, FreeSurfer^76^ and Connectome Workbench^77^ for inhomogeneity correction, and spatial and surface registration to a standardized space, that is, the study template space. EDNiX is a brain imaging processing pipeline tailored for diverse mammalian species, supporting anatomical MRI, fMRI and positron emission tomography scans. It offers flexibility to accommodate several imaging constraints and acquisition sequences, facilitating data processing from raw images to statistical analysis. Designed to enhance cross-species and developmental neuroimaging research, EDNiX has been validated using open-source datasets from multiple laboratories. EDNiX is detailed in a forthcoming article and is currently available on GitHub (https://github.com/garincle/EDNiX) and Zenodo (10.5281/zenodo.14801141) for reference. Using EDNiX, we created a study-specific template based on the average of the last T1 anatomical image (T1^last^) of each animal when registered in a common space using the anats_to_common function from Sammba-MRI^78,79^. A high-resolution NIH macaque template (NMT), as well as the CHARM^80^ and Subcortical Atlas of the Rhesus Macaque^81^ segmentations, were registered to the study template. Individual anatomical T1 images (T1^*n*^) were registered to their T1^last^ each T1^last^ was registered to the study template. The two movement parameters (T1^*n*^ to T1^last^ and T1^last^ to study template) were combined to register T1^*n*^ to the study template. Inversion of these movement parameters was used to register the NMT atlases to the individual T1^*n*^ images. The co-registrations of the NMT atlases to T1^*n*^ were individually inspected and corrected manually when necessary. The volumes (in mm^3^) were calculated using the 3dhistog function from AFNI. Surface and thickness were reconstructed based on previously computed brain segmentations and using the Connectome Workbench. Figures were produced using the Connectome Workbench and nilearn^82^. Surface, thickness and volume measurements of cortical and subcortical structures were performed on the hemisphere opposite to the one where recordings were performed.

### DTI preprocessing

DTI data were acquired in pairs with a reverse-phase encoding direction in the second scan (for example, posterior-anterior versus anterior-posterior). A diffusion-weighted spin-echo echo-planar imaging sequence was used to obtain 82 whole-brain slices of 2-mm thickness in 30 directions. Data were processed for analysis using MRtrix3 (ref. ^83^) and the Oxford Centre of fMRI of the Brain Software Library (FSL). The raw Digital Imaging and Communications in Medicine (DICOM) images acquired from the scanner were converted to Neuroimaging Informatics Technology Initiative (NIFTI) format using dcm2nii; the corresponding bval and bvec files containing information pertinent to the diffusion gradient were combined across scans. Images were then denoised (dwidenoise) and mean *b* = 0 images were calculated. After this, susceptibility-induced and eddy current distortion was corrected using FSL (TOPUP) and (eddycorrect), respectively. A tensor model was fitted to each voxel (dwi2tensor) and FA maps were calculated (tensor2metric). A mask was also created from the T1-weighted image (bet), segmenting the brain and non-brain tissue from the whole head.

Each FA image was then registered to the animal’s respective skullstripped T1-weighted image by affine transformation (flirt; Extended Data Fig. 10). These co-registered images were subsequently registered to a diffusion-tensor-based white matter atlas for rhesus macaques^84^. This allowed for a group analysis of several parameters: (1) the directionality of water diffusion within white matter tissue (FA); (2) the mean apparent diffusion coefficient of the diffusion tensor (MD); (3) the principal eigenvalue or diffusion parallel to the principal axis of diffusion (axial diffusivity (AD)); and (4) the mean of the two non-principal eigenvalues, or the diffusion perpendicular to the principal axis of diffusion (RD). The MD, AD and RD maps were derived from the diffusion tensor using the same method used for generating the FA maps.

After initial processing, a whole-brain ROI analysis was performed, with 53 white matter tracts from the DTI atlas selected. Selected ROIs from the white matter atlas analyzed are described in Supplementary Table 10. The left and right hemispheres were calculated separately and averaged together.

### Behavioral analyses

We analyzed task performance in the ODR and distractor tasks as the percentage of trials that resulted in correct responses and by determining the animals’ saccade precision and RT. For the saccade precision analysis, we calculated each session’s saccade DI, defined as the area within one s.d. from the average landing position of each target condition^85^. We calculated RTs by determining the interval between the offset of the fixation point and the time of saccade onset.

### Firing metrics

We measured three metrics of neuronal firing from different task epochs using correct trials in the ODR tasks—firing rate, Fano factor and CV of firing rate and ISI—to characterize the intrinsic firing pattern of neurons recorded at different maturation stages. Firing metrics were calculated for each time point, which involved all the neurons recorded from one animal at one session. The firing rate measures the activity of the neurons; the Fano factor and CVs measure the variability of the neural activities. The CV of ISI is defined as:

$${\mathrm{CV}}_{\mathrm{ISI}}=\frac{{\sigma }_{\mathrm{ISI}}}{{\mu }_{\mathrm{ISI}}}$$

where ${\sigma }_{\mathrm{ISI}}$ is the s.d. of the ISIs and ${\mu }_{\mathrm{ISI}}$ is the mean of the ISIs. Similarly, the CV of the firing rate is defined as:

$${\mathrm{CV}}_{\mathrm{rate}}=\frac{{\sigma }_{\mathrm{rate}}}{{\mu }_{\mathrm{rate}}}$$

where ${\sigma }_{\mathrm{rate}}$ is the s.d. of the firing rate at baseline across all included trials and ${\mu }_{\mathrm{rate}}$ is the mean of the baseline firing rate. The Fano factor was computed as:

$$\mathrm{Fano}\,\mathrm{factor}=\frac{{{\sigma }^{2}}_{\mathrm{rate}}}{{\mu }_{\mathrm{rate}}}$$

We computed the Fano factor of spike counts to measure the variance of the firing rate as in previous studies^86^. Briefly, data for each condition in each neuron are initially treated separately. For each condition, we computed the variance (across trials) and mean of the firing rate during the epoch of interest. The Fano factor was the slope of the regression relating the variance to the mean.

### Single-neuron tuning

To investigate single-neuron tuning, we used firing rates from eight locations and fitted a Gaussian curve to the data. We defined the locations and corresponding firing rates. The Gaussian model function used to describe the tuning curve was:

$$f\left(\beta ,\mathrm{loc}\right)={\beta }_{1}+{\beta }_{2}\exp \left(-0.5{\left(\frac{\mathrm{loc}-{\beta }_{3}}{{\beta }_{4}}\right)}^{2}\right)$$

where *β*_1_ is the baseline firing rate, *β*_2_ is the amplitude of the Gaussian peak, *β*_3_ is the location of the peak and *β*_4_ is the s.d. (width) of the Gaussian curve. The lsqcurvefit function in MATLAB was used to fit the Gaussian model.

### Intrinsic timescales

To calculate the intrinsic timescale of single neurons, we constructed a data matrix where each row represented a trial and each column represented time bins for a single neuron, with a bin size of 50 ms. We used the 1,000 ms pre-cue fixation epochs of each neuron. We excluded neurons with an average firing rate of less than one spike per second and those with any time bins containing zero spikes across all trials. Neurons with poor fitting quality (for example, intrinsic timescales greater than 0.5 s) were also excluded from the final analysis^37,87^. The average autocorrelation was calculated for each time lag and normalized by the total variance of the spike counts. We then fitted an exponential decay model to the autocorrelation data using:

$$R\left({{\tau }}\right)=A \exp \left(-\frac{x}{{{\tau }}}\right)+B$$

where *A* is the amplitude, *τ* is the intrinsic timescale and *B* is an offset parameter. The fit function in MATLAB was used to fit the exponential model.

### Information contained in single neurons

We used the bias-corrected percentage of the *ω*PEV statistic to estimate single-neuron selectivity to task conditions, that is, the locations of visual stimuli^88^. We calculated the *ω*PEV using the Measures of Effect Size (MES) toolbox of MATLAB, v.1.6.0.0, which calculates the amount of variance in the neurons’ firing rate explained by the location of the stimuli. *ω*PEV is defined as:

$${\omega} {\mathrm {PEV}}=\frac{{{\mathrm{SS}}}_{{\mathrm{between}}\,{\mathrm{groups}}}-{\rm{d}}.{\rm{f}}.\,\times \,{\mathrm{MSE}}}{{{\mathrm{SS}}}_{\mathrm{total}}+{\mathrm{MSE}}}$$

fspacewhere ${\mathrm{SS}}_{\mathrm{total}}$ denotes the total sum of squares, that is, the total variance, ${\mathrm{SS}}_{{\mathrm{between}}\,{\mathrm{groups}}}$ denotes the variance between groups, d.f. the degrees of freedom and MSE is the mean squared error.

### Temporal coding dimensionality

Firing metrics use averaged measures in certain time windows and do not reflect the consistency or dynamics of neural responses over time. Therefore, we used PCA to quantify the temporal coding stability of each individual neuron. We estimated the effective dimensionality (*N*_eff_) of the temporal state space of each neuron^37^. PCA was performed on the mean firing rate of trials in the same task condition across different time bins during the cue presentation, thereby quantifying the temporal variability of task-dependent firing. For each neuron, we then constructed a data matrix consisting of *n* trials × 10 time bins. For each trial, we computed the firing rate in each time bin. Each time bin is a 50-ms time in the 500-ms stimulus presentation. Then, we averaged the binned firing rates according to task condition (eight locations). The columns in the matrix correspond to ten adjacent, independent time windows spanning 500 ms and rows to the eight task condition averages. PCA was then performed on the matrix to quantify and arrange the variance along the principal components in the subspace spanned by the ten independent time bins. The eigenvalues associated with each principal component give us the means to quantify the effective dimensionality (*N*_eff_) of our temporal state space:

$${N}_{\mathrm{eff}}=\frac{{\left(\sum \lambda \right)}^{2}}{\sum {\lambda }^{2}}$$

where *λ* represents the eigenvalues.

### Dimensionality in full neural space across maturation intervals

Like the dimensionality in temporal space of each neuron, we calculated the dimensionality of neural responses in the full *N*-dimensional neural space (where *N* is the number of neurons) to test global changes in the neural space across maturation. For the population analysis, neurons from all animals were pooled and analyzed together. We segmented the neurons based on their mid-adolescence age into 20 even time intervals (each ~200 days long), spanning from the earliest to the latest session recorded across all animals. For each maturation interval, we combined neurons across animals to create a pseudo-population. We averaged the firing rate per condition (eight locations in the ODR task) within 200-ms bins, stepped by 100 ms. The average response was calculated for each condition in each time bin during the delay period of the task (1.5 s). To control for the effect of sample size on dimensionality, we randomly sampled 30 neurons from each pseudo-population to ensure no oversampling from any populations in each iteration. PCA was performed on the concatenated data: size = (condition × time) × 30 neurons. We then calculated the effective dimensionality (*N*_eff_) of each maturation interval. The distribution of *N*_eff_ values was estimated with a bootstrap: we resampled neurons with replacement per maturation interval 500 times and then performed the estimation of *N*_eff_. This gave us a dataset of 10,000 values of *N*_eff_ and these values were used to estimate the change in *N*_eff_ across adolescent development and were fitted with GAMM.

### Rotation of subspaces

We applied PCA to denoise and quantify the difference between representation of the target and distractor. PCA was performed on the mean firing rate of neurons across 15 maturation intervals (200 days) from four animals who performed the distractor task.

For each neuron, we calculated the average firing rate of the cue and distractor epochs in all correct trials for different task conditions (four locations). The firing rates of each neuron formed an 8 × 1 vector (four locations × two epochs). The population activity matrix size is 8 × *N* where *N* is the number of neurons in the population used. Then, we *z*-scored the population activity matrix to account for the baseline firing difference among neurons. To find the rotation between two periods, we applied PCA on the *z*-scored population matrix. We selected the first three eigenvectors of the covariance and sorted them according to decreasing order in terms of explaining the variance.

We then projected the population activity matrix into a three-dimensional PCA space. The population representations of each of the four spatial locations in two task epochs were represented by a vector. Four spatial locations in each of two task epochs then formed a subspace respectively spinning in the full space. We found the best-fit hyperplanes for the cue and distractor subspaces using least squares to minimize the distance from each point to the hyperplane, implemented using the fminsearch function in MATLAB. To examine the relationship between the two subspaces, the rotation was defined as the acute angle between the normal vectors of the two best-fit planes.

As with effective dimensionality, we randomly sampled 30 neurons from each of the 15 intervals to control the effect of sample size. The distribution of rotation angles was estimated using bootstrapping: we resampled neurons with a replacement per maturation interval 500 times and calculated the rotation angle in each resampling. The total dataset contained 7,500 values of rotation angles and was then used to estimate the change in rotation of subspaces across adolescent development and fitted with GAMMs.

### GAMMs

We sought to characterize the adolescent development of nonhuman primates in cognitive function, PFC activity and the brain regions and structures that matured in our longitudinal sample. We expected developmental stages to exhibit a nonlinear relationship with each outcome. Therefore, we used GAMMs. GAMM is a flexible, semiparametric method for identifying and estimating nonlinear effects of covariates on the outcome variable when observations are not independent^89^.

All GAMM analyses were implemented using the mgcv package for R (version 1.9-3) to fit a series of GAMMs for the outcomes of interest, each with a smooth function of mid-adolescence age as a covariate, using a thin plate regression spline basis to estimate this smooth function. The random effects in each GAMM included animal-specific intercepts and slopes for mid-adolescence age. For proportional outcomes like behavioral task performance, the quasi-binomial family and logit link functions were used. For all other outcomes, which are continuous, the Gaussian family and identity link functions were used. For those models for which there was a statistically significant fixed effect, the gratia package (version 0.11-1) for R^90^ was used to conduct exploratory post-hoc analyses to identify significant periods of developmental change. Specifically, the derivatives of each estimated smooth function of age were approximated using the method of finite differences and a simultaneous 95% confidence. Because we tested the effect of age on several outcome measures, in separate GAMMs, we applied a false discovery rate correction (Benjamini–Hochberg) to control for multiple comparisons. The reported *P* values reflect the adjusted significance levels unless otherwise specified.

### Correlation between trajectories

To evaluate the similarity between developmental trajectories, we calculated the correlation between the GAMM predictions of different measures. To ensure consistency and comparability, the predictor values (mid-adolescence age) were evenly sampled at 100 intervals between the earliest and latest time points for behavioral data. Each curve was then normalized using *z*-score normalization.

We calculated two key metrics: the Pearson correlation coefficient (*r*) and the RMSE. Before calculating the RMSE, each normalized curve was shifted such that the starting point was aligned to zero. This was done by subtracting the starting value of the curve. After shifting, the absolute values of the data points were taken to ensure uniformity in the direction of both curves, facilitating a comparable visualization using RMSE between positively and negatively correlated pairs of curves. The RMSE was calculated as:

$$\mathrm{RMSE}=\sqrt{\frac{1}{n}\mathop{\sum }\limits_{i=1}^{n}{\left(\left|{X}_{i}\right|-\left|{Y}_{i}\right|\right)}^{2}}$$

where $| {X}_{i}|$ and $| {Y}_{i}|$ are the absolute values of the shifted and normalized data points from the respective curves.

We conducted a permutation test with a maxT approach to assess the statistical significance of the observed correlation coefficients. We randomly permuted *X* and *Y* to recalculate the correlation coefficient across 1,000 permutations to simulate the null hypothesis of no correlation. The maxT *P* value was calculated to determine the probability of observing a correlation as extreme as the one detected, or more extreme, under the null hypothesis.

### MERF

As an alternative to obtaining correlation measures between fitted trajectories of behavioral, neural and brain structural trajectories, we also directly modeled the raw values of the measures in a unified framework using MERF. Our primary behavioral measures were DI and RT. We included the volume of brain segmentations and structures, the FA of white matter tracts and the neuron firing metrics as input factors. When necessary, we used animal-specific predictions from our GAMM fits (including random intercepts) to align the measures with the time points of the behavioral performance data similarly to the correlation analysis.

MERF extends the standard random forest approach by allowing for both fixed (the predictors of interest) and random (animal-level intercepts or slopes) effects. This is particularly relevant given the longitudinal structure of our data. We used a Python-based MERF implementation. Animal ID was specified as a random effect to account for individual differences. All other inputs served as predictors in the fixed-effects portion of the model. The number of trees (*n*_estimators) was set at 100 and minimum samples per split (min_samples_split) was two during the training.

We used SHAP values to quantify each predictor’s contribution to a given prediction. SHAP provides a consistent and theoretically justified measure of feature contribution. For each data point, we obtained SHAP values that showed how much each predictor increased or decreased the model’s prediction relative to a baseline. We aggregated SHAP values across all animals and time points to rank variables according to their median absolute contribution.

### Reporting summary

Further information on research design is available in the Nature Portfolio Reporting Summary linked to this article.

## Online content

Any methods, additional references, Nature Portfolio reporting summaries, source data, extended data, supplementary information, acknowledgements, peer review information; details of author contributions and competing interests; and statements of data and code availability are available at 10.1038/s41593-025-02076-0.

## Supplementary information

Supplementary Information Supplementary Figs. 1–10 and Tables 1–10.

Reporting Summary

## Source data

Source data Figs. 1–6 and Extended Data Figs. 1 and 3–9 Source data for Figs. 1–6 and Extended Data Figs. 1 and 3–9.

## Data Availability

Data for the current study are available through at Zenodo 10.5281/zenodo.14832030. Source data are provided with this paper.

## Code availability

The code used to process the results and generate the figures is available at CodeOcean: https://codeocean.com/capsule/7858389/tree. The pipeline to process the MRI anatomical images is available at GitHub https://github.com/garincle/EDNiX and Zenodo 10.5281/zenodo.14801141.
