# Supplementary material for: Longitudinal measures of monkey brain structure and activity through adolescence predict cognitive maturation
Source: Nat Neurosci. 2025 Oct 27;28(11):2344–55. doi: 10.1038/s41593-025-02076-0 (PMC12586175; doi:10.1038/s41593-025-02076-0)
Supplement: Supplementary file 1 — Supplementary Figs. 1–10 and Tables 1–10. [file 41593_2025_2076_MOESM1_ESM.pdf]

nature neuroscience

Supplementary information

https://doi.org/10.1038/s41593-025-02076-0

Longitudinal measures of monkey brain structure and activity through adolescence predict cognitive maturation

In the format provided by the authors and unedited

Supplementary Information

Contents

- **Supplementary Tables**
- **Supplementary Figures**

Supplementary Tables

Supplementary Table 1

Measures	Variable	edf	F	P value	AIC
Body weight	Chronological age	3.14	22.14	$<10^{-6}$	834.10
	mid-adolescence age + sex	3.29	13.26	$<10^{-6}$	822.50
	**mid-adolescence age**	5.67	26.77	$<10^{-6}$	**792.43**
Canine length	Chronological age	1.00	153.85	$<10^{-6}$	1054.95
	mid-adolescence age + sex	3.29	11.60	$<10^{-6}$	1040.20
	**mid-adolescence age**	4.51	39.20	$<10^{-6}$	**1035.80**
Femur length	Chronological age	6.27	29.68	$<10^{-6}$	753.19
	**mid-adolescence age + sex**	3.97	8.31	5.97×10^{-6}	**751.01**
	mid-adolescence age	5.72	13.78	$<10^{-6}$	771.09
Nipple length	Chronological age	4.16	12.88	4.00×10^{-6}	260.70
	mid-adolescence age + sex	4.03	11.05	1.51×10^{-5}	260.70
	**mid-adolescence age**	4.03	11.05	1.51×10^{-5}	**260.32**
Testis length	Chronological age	3.55	26.73	$<10^{-6}$	677.16
	mid-adolescence age + sex	5.89	63.84	$<10^{-6}$	677.16
	**mid-adolescence age**	5.89	63.84	$<10^{-6}$	**648.43**
Testis volume	Chronological age	4.04	88.10	$<10^{-6}$	808.09
	mid-adolescence age + sex	5.89	63.84	$<10^{-6}$	808.09
	**mid-adolescence age**	5.40	97.21	$<10^{-6}$	**780.90**
Trunk length	Chronological age	5.16	23.23	$<10^{-6}$	692.97
	**mid-adolescence age + sex**	3.72	19.14	$<10^{-6}$	**685.51**
	mid-adolescence age	5.84	34.28	$<10^{-6}$	688.83

Supplementary Table 1. Morphometric measures and model fit parameters aligned on chronological age, mid-adolescence age, and mid-adolescence age including an additional sex covariate. The model with the lowest AIC score appears in bold font, for each measure.

Supplementary Table 2

subject	ρ.c	s.shift	l.shift
O	0.964	0.963	-0.210
P	0.936	0.744	-0.130
Q	1.000	1.010	-0.012
R	0.990	1.057	0.132
S	0.995	1.045	-0.028
T	0.899	0.895	-0.424
U	0.978	1.194	-0.101
V	0.787	0.989	0.509
**mean**	**0.944**	**0.987**	**-0.033**

Supplementary Table 2. Leave-one-monkey-out (LOO) analysis for dispersion index (DI) in the ODR task. Results were obtained by re-fitting the model on the data excluding that monkey and then comparing the prediction curve over the mid-adolescence age grid to the full model's predictions using the Concordance Correlation Coefficient (CCC). Overall agreement (ρ.c), scale shift (s.shift), and location shift (l.shift) for DI are shown.

Supplementary Table 3

subject	ρ.c	s.shift	l.shift
OLI	0.918	1.250	-0.012
PIC	0.993	1.036	0.100
QUA	0.997	0.983	-0.077
ROS	0.985	0.942	-0.091
SON	0.993	0.896	-0.032
TRI	0.973	0.976	0.130
UNI	0.992	1.001	0.007
VIK	0.984	0.927	0.039
mean	0.979	1.001	0.008

Supplementary Table 3. Leave-one-monkey-out (LOO) analysis for reaction (RT) time in the ODR task, calculated as in Supplementary Table 5. Overall agreement (ρ.c), scale shift (s.shift), and location shift (l.shift) for RT calculated in the ODR task.

Supplementary Table 4

Subject	N (neurons)	N (sessions)
O	292	53
P	264	52
Q	62	16
R	236	56
S	330	61
T	262	36
U	527	75
V	158	38

Supplementary Table 4. Sample sizes for neurons and sessions in each subject.

Supplementary Table 5

subject	ρ.c	s.shift	l.shift
O	0.772	0.785	0.346
P	0.767	0.695	0.014
Q	0.824	0.915	0.224
R	0.832	0.818	0.139
S	0.850	1.118	0.078
T	0.901	1.070	-0.186
U	0.812	0.809	0.245
V	0.813	0.854	0.254
**mean**	**0.821**	**0.883**	**0.139**

Supplementary Table 5. Leave-one-monkey-out (LOO) analysis for baseline firing rate in the ODR task, calculated as in Supplementary Table 5. Overall agreement (ρ.c), scale shift (s.shift), and location shift (l.shift) for baseline firing rate calculated in the ODR task.

Supplementary Table 6

subject	ρ.c	s.shift	l.shift
O	0.997	0.931	0.028
P	0.997	0.968	-0.050
Q	0.999	0.967	0.019
R	0.984	0.997	0.176
S	0.890	0.641	-0.218
T	0.984	1.175	-0.044
U	0.997	1.059	0.000
V	0.993	1.118	0.035
**mean**	**0.980**	**0.982**	**-0.007**

Supplementary Table 6. Leave-one-monkey-out (LOO) analysis for Fano factor of spike counts in the ODR task, calculated as in Supplementary Table 5. Overall agreement (ρ.c), scale shift (s.shift), and location shift (l.shift) for Fano factor in the ODR task.

Supplementary Table 7

subject	ρ.c	s.shift	l.shift
O	0.981	0.959	0.190
P	0.873	0.650	0.267
Q	0.994	0.973	0.094
R	0.986	0.958	-0.150
S	0.991	1.005	-0.100
T	0.996	0.953	0.047
U	0.989	1.002	0.069
V	0.997	1.018	-0.078
**mean**	**0.976**	**0.940**	**0.042**

Supplementary Table 7. Leave-one-monkey-out (LOO) analysis for temporal dimensionality, calculated as in Supplementary Table 5. Overall agreement (ρ.c), scale shift (s.shift), and location shift (l.shift) for temporal dimensionality.

Supplementary Table 8

Input Factors on DI	Sum of SHAP
FA_cerebellar_peduncle_contra	928.890
FA_CST_contra	786.151
FA_MLF_contra	342.088
FA_retrolenticular_LIC_contra	306.436
FA_ALIC_contra	265.556
FA_body_cc	252.496
orbital_frontal_cortex_contra	167.576
FA_SCP_contra	157.434
FA_midbrain_contra	120.295
FA_cingulum_bundle	108.062
FA_STG_contra	105.165
baseline firing rate	94.009
FA_PCT	80.763
FA_splenium	80.552
FA_PCR_contra	79.781
FA_fornix	55.747
FA_SLF_contra	55.555
FA_external_capsule_contra	52.864
FA_dorsal_PCR_contra	46.626
FA_PLIC_contra	30.885
FA_ACR_contra	27.820
FA_ICP_contra	25.446
FA_uncinate_fasciculus_contra	23.366
FA_CgC_contra	22.473
FA_SFOF_contra	20.387
SC_contra.x	19.122
metencephalon_contra	18.34
FA_MTG_contra	18.055
FA_superior_cingulum_contra	17.847
FA_anterior_cingulum_contra	16.562
SC_contra.y	13.184
FA_thalamus_contra	12.216
Cerebral_White_Matter_contra	11.052
FA_IFG_contra	10.65
FA_SCR_contra	9.395
diencephalon_contra	8.837
FA_PTR_contra	8.638
FA_perihippocampal_cingulum_contra	7.628

mesencephalon_contra	7.546
CSF	6.092
FA_genu	5.992
motor_cortex_contra	5.062
lateral_prefrontal_cortex_contra	4.569
anterior_cingulate_gyrus_contra	4.538
Temporal_contra	3.787
Frontal_contra	3.366
Parietal_contra	3.159
Occipital_contra	3.088
myelencephalon_contra	2.994
Cerebral_Cortex_contra	2.908

Supplementary Table 8. Results from the Mixed-Effects Random Forest (MERF) predicting Dispersion Index (DI). The table lists predictors ranked by aggregated absolute SHAP values, which quantify the relative contribution of each predictor to the model. Greater SHAP values indicate stronger predictive influence on behavioral performance (DI).

Supplementary Table 9

Input factors of RT	sum of SHAP
FA_CgC_contra	6.192
FA_cingulum_bundle	5.646
baseline firing rate	5.503
motor_cortex_contra	4.741
FA_STG_contra	1.854
Cerebral_Cortex_contra	0.963
orbital_frontal_cortex_contra	0.842
FA_fornix	0.694
FA_SFOF_contra	0.533
anterior_cingulate_gyrus_contra	0.522
FA_IFG_contra	0.255
FA_dorsal_PCR_contra	0.216
FA_midbrain_contra	0.176
SC_contra.y	0.123
SC_contra.x	0.114
FA_PCT	0.109
FA_superior_cingulum_contra	0.107
FA_thalamus_contra	0.102
FA_MLF_contra	0.101
FA_SLF_contra	0.098
FA_perihippocampal_cingulum_contra	0.096
CSF	0.093
FA_retrolenticular_LIC_contra	0.075
FA_ICP_contra	0.065
FA_external_capsule_contra	0.065
FA_CST_contra	0.060
FA_SCR_contra	0.060
FA_PTR_contra	0.058
FA_MTG_contra	0.055
FA_anterior_cingulum_contra	0.055
FA_ACR_contra	0.054
metencephalon_contra	0.046
FA_uncinate_fasciculus_contra	0.043
Frontal_contra	0.042
mesencephalon_contra	0.041
FA_PCR_contra	0.040
FA_genu	0.039
Parietal_contra	0.038

FA_SCP_contra	0.035
FA_ALIC_contra	0.034
FA_body_cc	0.033
Temporal_contra	0.030
lateral_prefrontal_cortex_contra	0.029
FA_splenium	0.028
FA_cerebellar_peduncle_contra	0.027
Occipital_contra	0.026
myelencephalon_contra	0.026
FA_PLIC_contra	0.023
Cerebral_White_Matter_contra	0.023
diencephalon_contra	0.022

Supplementary Table 9. Results from the Mixed-Effects Random Forest (MERF) predicting Reaction Time (RT). Similar to Supplementary Table 10, predictors are ranked by absolute SHAP values, with higher values representing greater importance in predicting RT.

Supplementary Table 10

Regions - Hemisphere	Connection	Abbreviation
Anterior Limb of the Internal Capsule - Left	Thalamus and Frontal Cortical	ALIC L
Anterior Limb of the Internal Capsule - Right		ALIC R
Anterior Cingulum WM - Left	Hippocampus, Parietal, and Frontal	anterior cingulum L
Anterior Cingulum WM - Right		anterior cingulum R
Anterior Corona Radiata - Left	Internal Capsule and Cortical	ACR L
Anterior Corona Radiata - Right		ACR R
Body of Corpus Callosum	Interhemispheric Sensorimotor/Posterior	body cc
Cerebellar Peduncle - Left	Brainstem and Internal Capsule	cerebellar peduncle L
Cerebellar Peduncle - Right		cerebellar peduncle R
Corticospinal Tract - Left	Sensorimotor	CST L
Corticospinal Tract - Right		CST R
Dorsal Posterior Corona Radiata - Left	Internal Capsule and Cortical	dorsal PCR L
Dorsal Posterior Corona Radiata - Right		dorsal PCR R
External Capsule - Left	Frontal, Parietal, Occipital, and Temporal	external capsule L
External Capsule - Right		external capsule R
Fornix	Hippocampus and Septal Nuclei	fornix
Genu of Corpus Callosum	Interhemispheric Frontal	genu
Inferior Cerebellar Peduncle - Left	Cerebellar Input	ICP L
Inferior Cerebellar Peduncle - Right		ICP R
Inferior Frontal Gyrus WM - Left		IFG L
Inferior Frontal Gyrus WM - Right		IFG R
Middle Temporal Gyrus WM - Left		MTG L
Middle Temporal Gyrus WM - Right		MTG R
Midbrain White Matter WM - Left		midbrain L
Midbrain White Matter WM - Right		midbrain R
Middle Longitudinal Fasciculus - Left	Frontal, Parietal, Occipital, and Temporal	MLF L
Middle Longitudinal Fasciculus - Right		MLF R
Perihippocampal Cingulum - Left	Hippocampus, Parietal, and Frontal	perihippocampal cingulum L
Perihippocampal Cingulum - Right		perihippocampal cingulum R
Posterior Limb of the Internal Capsule – Left	Corticospinal and Thalamocortical	PLIC L
Posterior Limb of the Internal Capsule - Right		PLIC R
Pontine Crossing Tract	Spinal Cord and Cerebellar Crossing Fibers	PCT
Posterior Corona Radiata - Left	Internal Capsule and Cortical	PCR L
Posterior Corona Radiata - Right		PCR R
Posterior Thalamic Radiation - Left	Optic Radiation	PTR L
Posterior Thalamic Radiation - Right		PTR R
Retrolenticular Limb of the Internal Capsule - Left	Thalamus and Posterior Cortical	retrolenticular LIC L
Retrolenticular Limb of the Internal Capsule - Right		retrolenticular LIC R
Superior Fronto-Occipital Fasciculus - Left	Frontal, Parietal, and Occipital	SFOF L
Superior Fronto-Occipital Fasciculus - Right		SFOF R
Superior Longitudinal Fasciculus - Left	Frontal, Parietal, Occipital, and Temporal	SLF L
Superior Longitudinal Fasciculus - Right		SLF R

Splenium of Corpus Callosum	Interhemispheric Posterior	splenium
Superior Cerebellar Peduncle - Left	Cerebellar Output	SCP L
Superior Cerebellar Peduncle - Right		SCP R
Superior Cingulum - Left	Hippocampus, Parietal, and Frontal	superior cingulum L
Superior Cingulum - Right		superior cingulum R
Superior Corona Radiata - Left	Internal Capsule and Cortical	SCR L
Superior Corona Radiata - Right		SCR R
Superior Temporal Gyrus WM - Left		STG L
Superior Temporal Gyrus WM - Right		STG R
Uncinate Fasciculus - Left	Hippocampus and Orbitofrontal Cortex	uncinate fasciculus L
Uncinate Fasciculus - Right		uncinate fasciculus R

Supplementary Table 10. Description of selected ROIs from white matter (WM) atlas analyzed.

Supplementary Figures

Supplementary Fig. 1

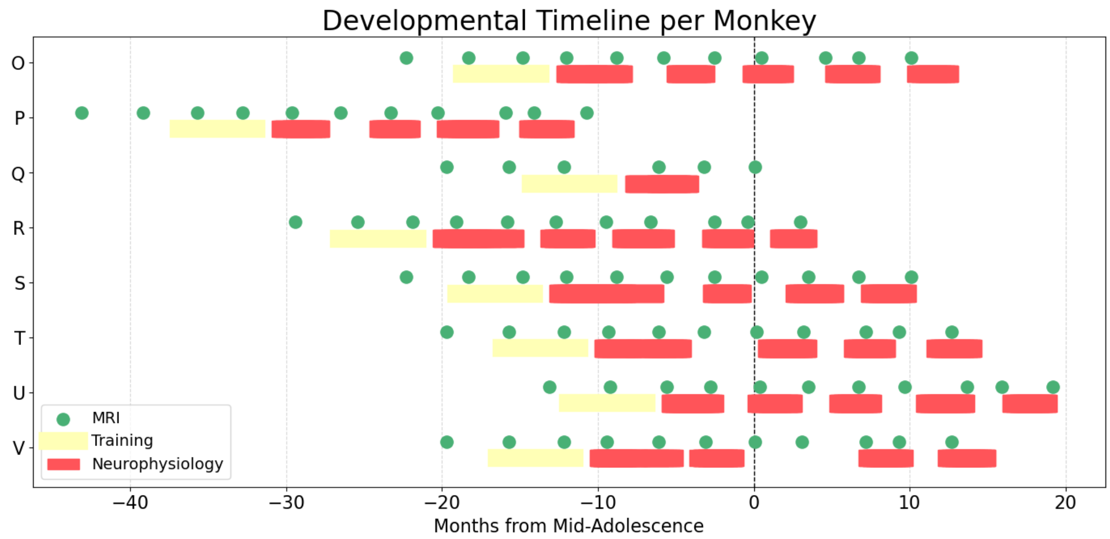

Supplementary Fig. 1. Developmental timeline for each subject. Each horizontal line represents an individual subject, aligned relative to their estimated mid-adolescence age (0 months, indicated by the vertical dashed line). Green dots mark the ages at which MRI scans were performed, yellow bars denote the period of task training (spanning from 6 months to 1 month before the initial task session), and red bars indicate the periods at which task performance assessments were conducted.

Supplementary Fig. 2

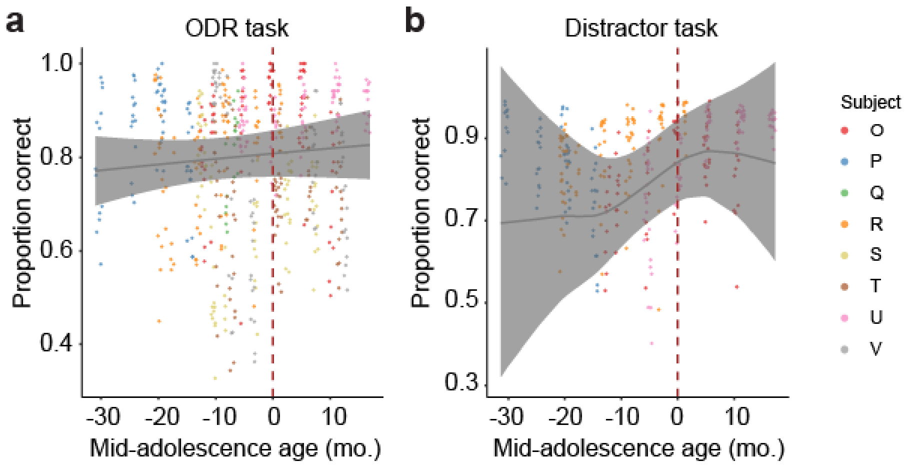

Supplementary Fig. 2. Trial completion performance. Percentage of trials that were deemed correct and rewarded as a function of age. (a) Performance in the oculomotor delayed response (ODR) task. (b) Performance in the ODR with distractor task. The dashed vertical line denotes the mid-adolescence age 0. Gray curves indicate non-significant changes along mid-adolescence age.

Supplementary Fig. 3

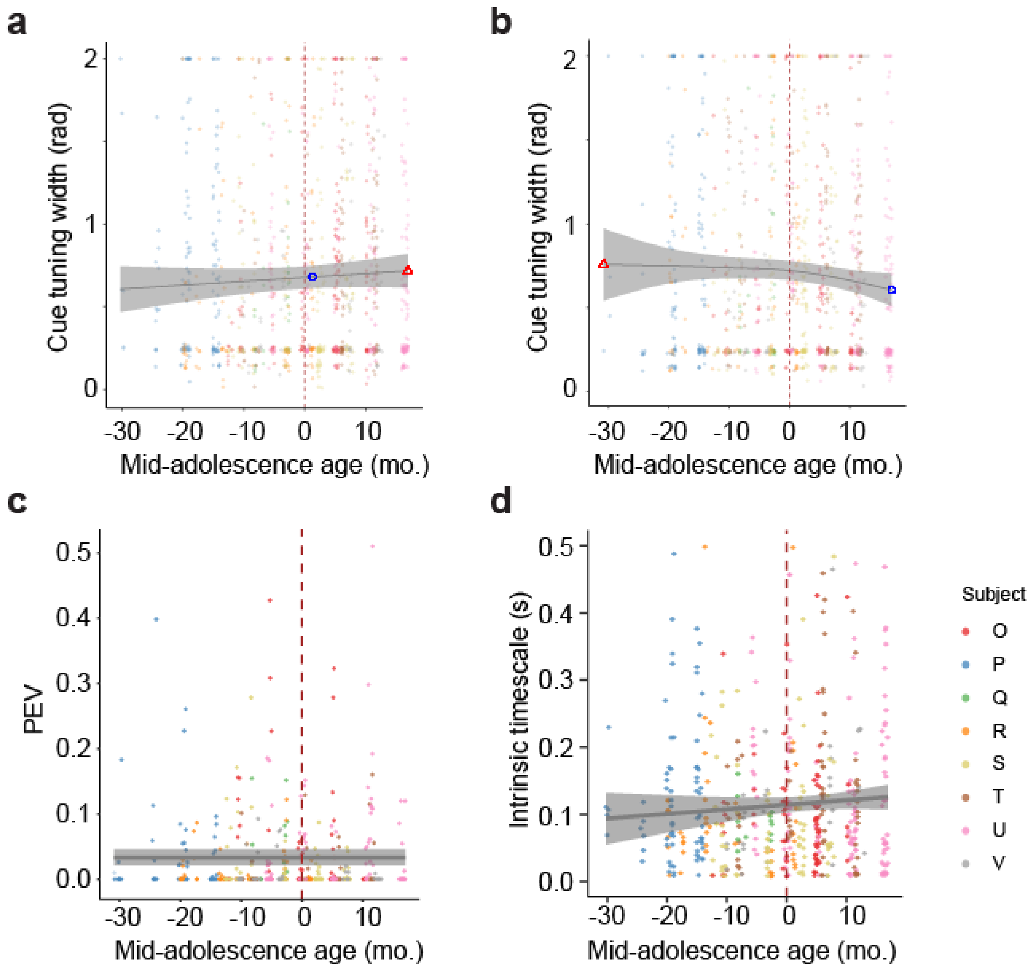

Supplementary Fig. 3. Maturation of neuronal tuning and intrinsic timescale. (a) Tuning width estimated during the cue period as a function of time relative to the mid-adolescence age. Gray line showed the GAMM fitted trajectory. Gray shaded regions denote the 95% confidence intervals (CIs). The dashed vertical line denotes the mid-adolescence age 0. (b) As in a, for tuning during the delay period of the ODR task. (c) Percentage of explained variance (ω^2) of each neuron during cue epoch of ODR task. (d) Intrinsic timescale as a function of time relative to the mid-adolescence age, each dot is one neuron (n = 508).

Supplementary Fig. 4

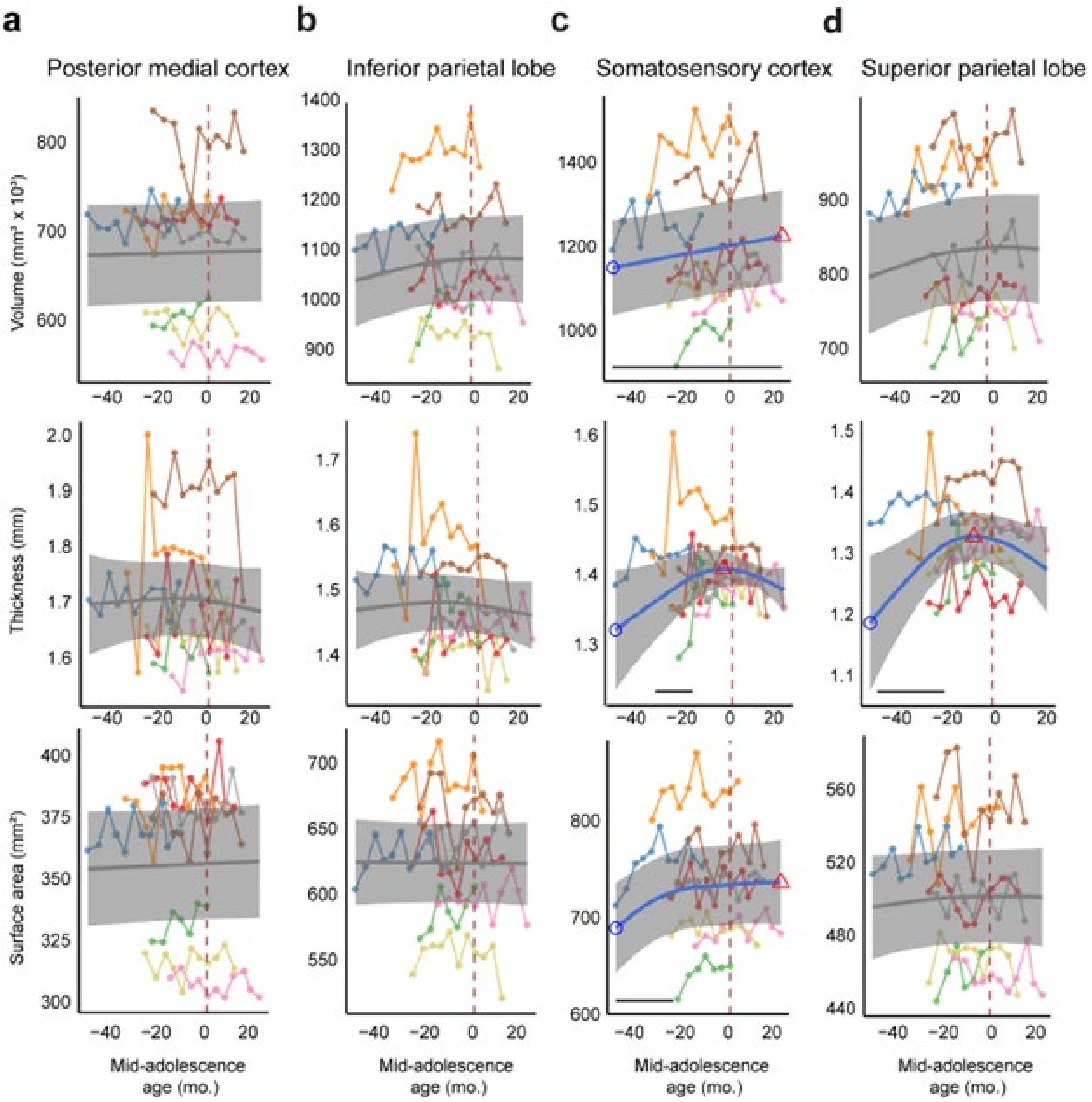

Supplementary Fig. 4. Raw data and fitted structural developmental trajectories of areas in parietal lobe. (a-d) Volume, thickness and surface area of the areas in parietal lobe as a function of age. In each panel, top: cortical volume; middle: cortical thickness; bottom: surface area. ROIs were determined using Charm atlas level 2. Blue or gray curve indicates the GAMM fitted trajectory. Gray shaded regions denote the 95% confidence intervals (CIs). Blue circle denotes the time of peak development velocity. Red triangle denotes the time of maximum value. Dashed vertical line denotes the mid-adolescence age 0. The horizontal bar denotes significant developmental effect intervals.

Supplementary Fig. 5

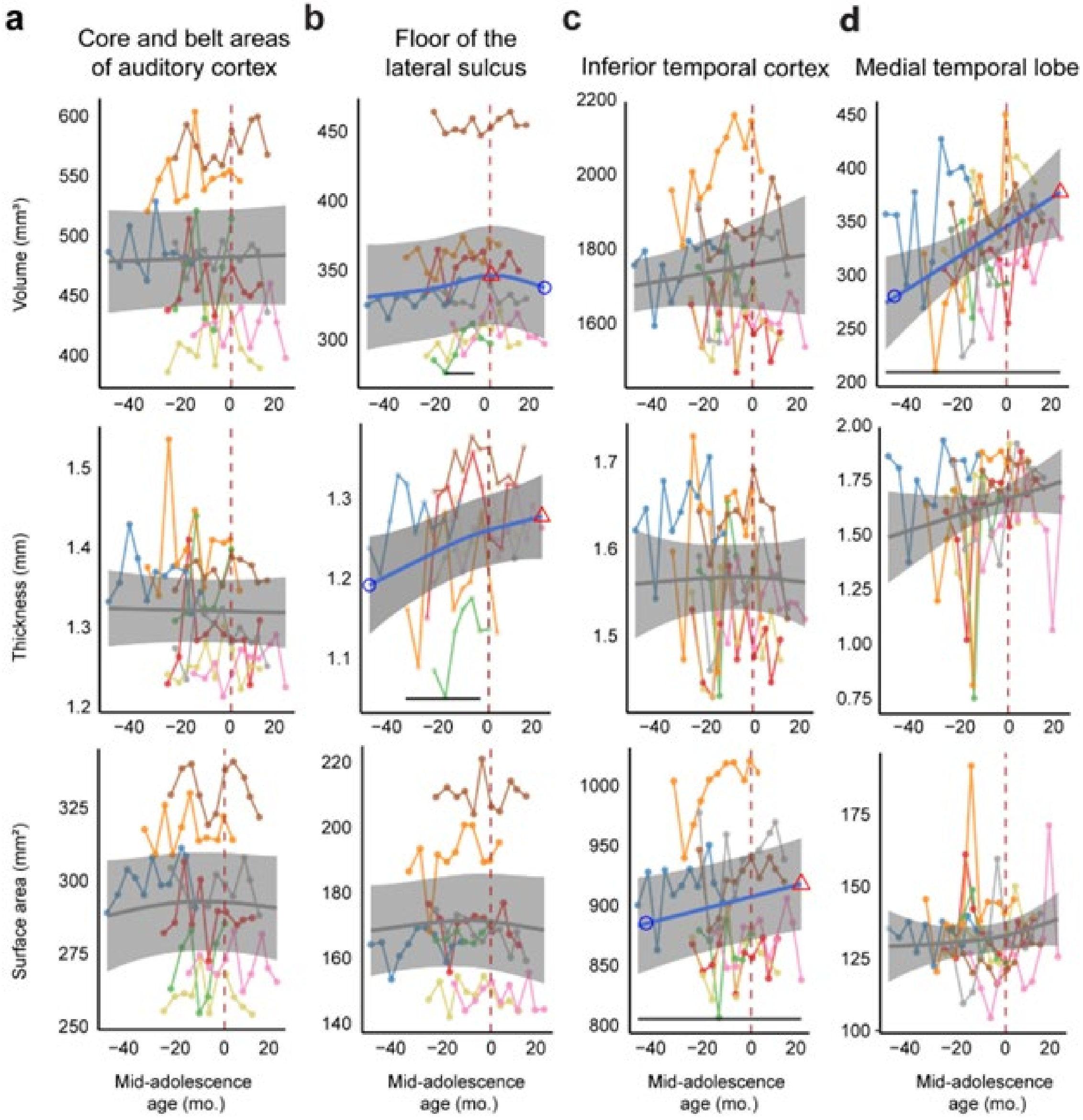

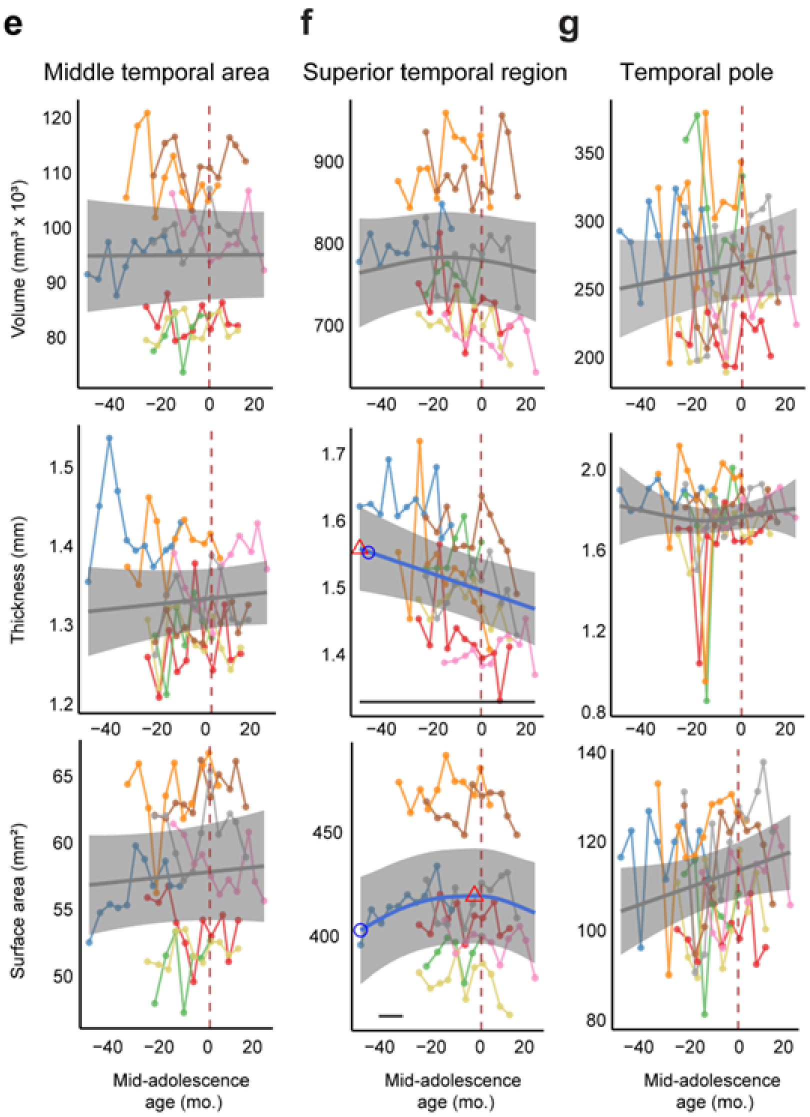

Supplementary Fig. 5. Raw data and fitted structural developmental trajectories of areas in temporal lobe. (a-g) Volume, thickness and surface area of the areas in temporal lobe as a function of age. In each panel, top: cortical volume; middle: cortical thickness; bottom: surface area. ROIs were determined using Charm atlas level 2. Blue or gray curve indicates the GAMM fitted trajectory. Gray shaded regions denote the 95% confidence intervals (CIs). Blue circle denotes the time of peak development velocity. Red triangle denotes the time of maximum value. Dashed vertical line denotes the mid-adolescence age 0. The horizontal bar denotes significant developmental effect intervals.

Supplementary Fig. 6

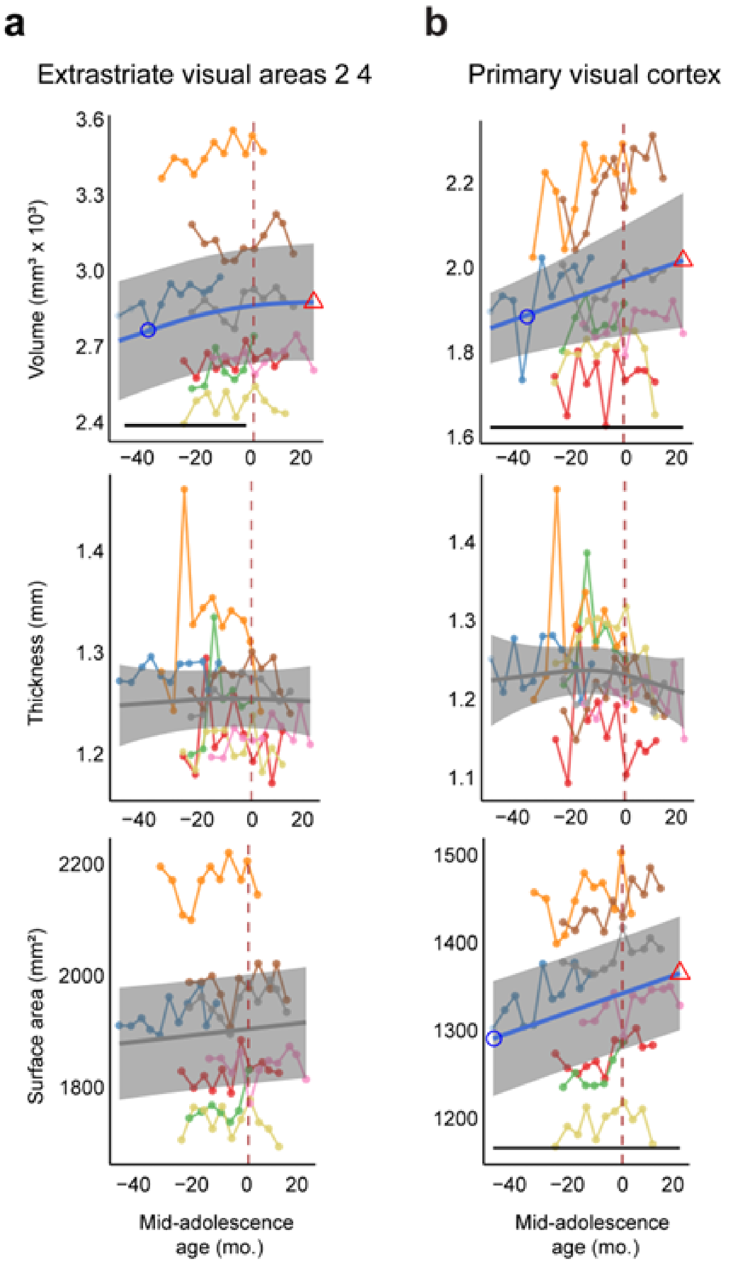

Supplementary Fig. 6. Raw data and fitted structural developmental trajectories of areas in occipital lobe. (a-d) Volume, thickness and surface area of the areas in occipital lobe as a function of age. In each panel, top: cortical volume; middle: cortical thickness; bottom: surface area. ROIs were determined using Charm atlas level 2. Blue or gray curve indicates the GAMM fitted trajectory. Gray shaded regions denote the 95% confidence intervals (CIs). Blue circle denotes the time of peak development velocity. Red triangle denotes the time of maximum value. Dashed vertical line denotes the mid-adolescence age 0. The horizontal bar denotes significant developmental effect intervals.

Supplementary Fig. 7

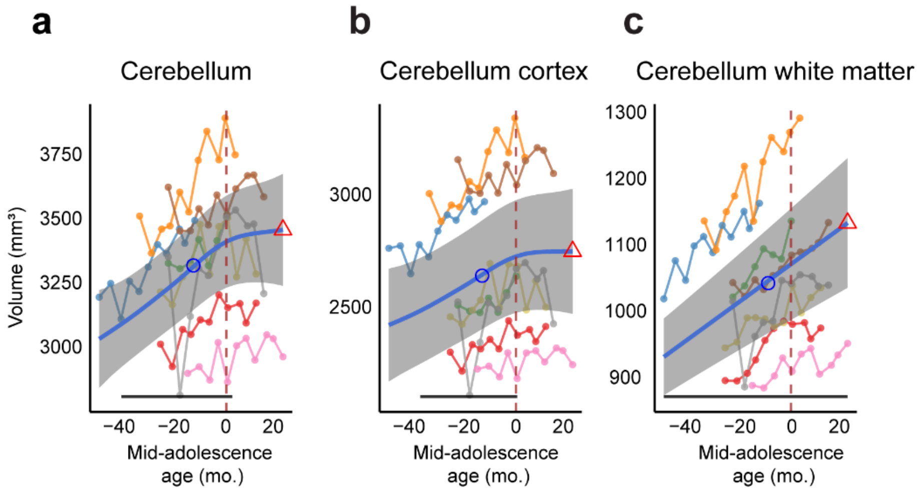

Supplementary Fig. 7. Raw data and fitted volumetric developmental trajectories of cerebellum. (a-c) Volume of cerebellum, cerebellum cortex and cerebellum white matter as a function of age. In each panel. Blue curve indicates the GAMM fitted trajectory. Gray shaded regions denote the 95% confidence intervals (CIs). Blue circle denotes the time of peak development velocity. Red triangle denotes the time of maximum value. Dashed vertical line denotes the mid-adolescence age 0. The horizontal bar denotes significant developmental effect intervals.

Supplementary Fig. 8

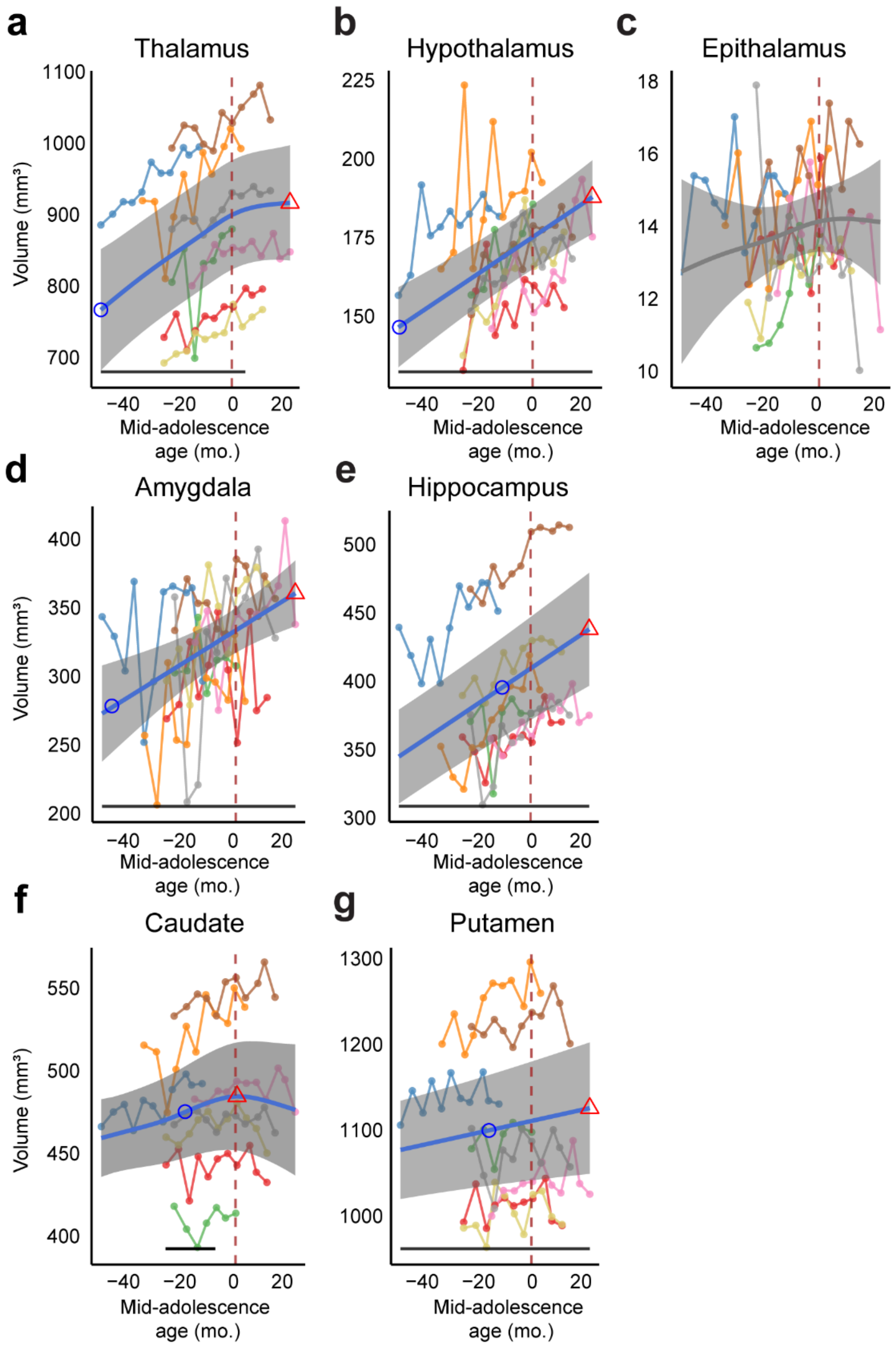

Supplementary Fig. 8. Raw data and fitted volumetric developmental trajectories of subcortical regions. Blue curve indicates the GAMM fitted trajectory. Gray shaded regions denote the 95% confidence intervals (CIs). Blue circle denotes the time of peak development velocity. Red triangle denotes the time of maximum value. Dashed vertical line denotes the mid-adolescence age 0. The horizontal bar denotes significant developmental effect intervals.

Supplementary Fig. 9

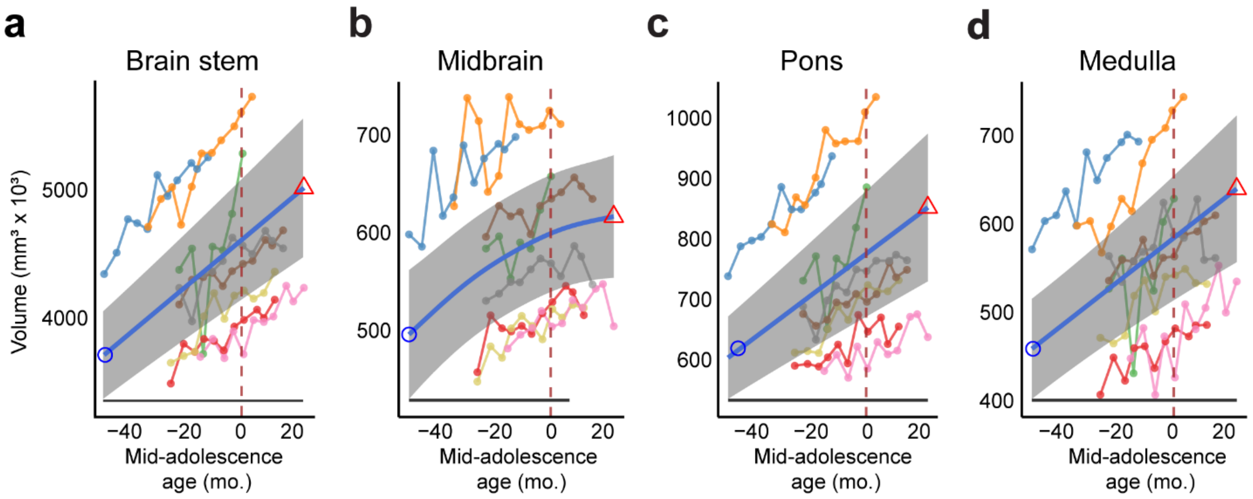

Supplementary Fig. 9. Raw data and fitted volumetric developmental trajectories of Brain stem structures. Blue curve indicates the GAMM fitted trajectory. Gray shaded regions denote the 95% confidence intervals (CIs). Blue circle denotes the time of peak development velocity. Red triangle denotes the time of maximum value. Dashed vertical line denotes the mid-adolescence age 0. The horizontal bar denotes significant developmental effect intervals.

Supplementary Fig. 10

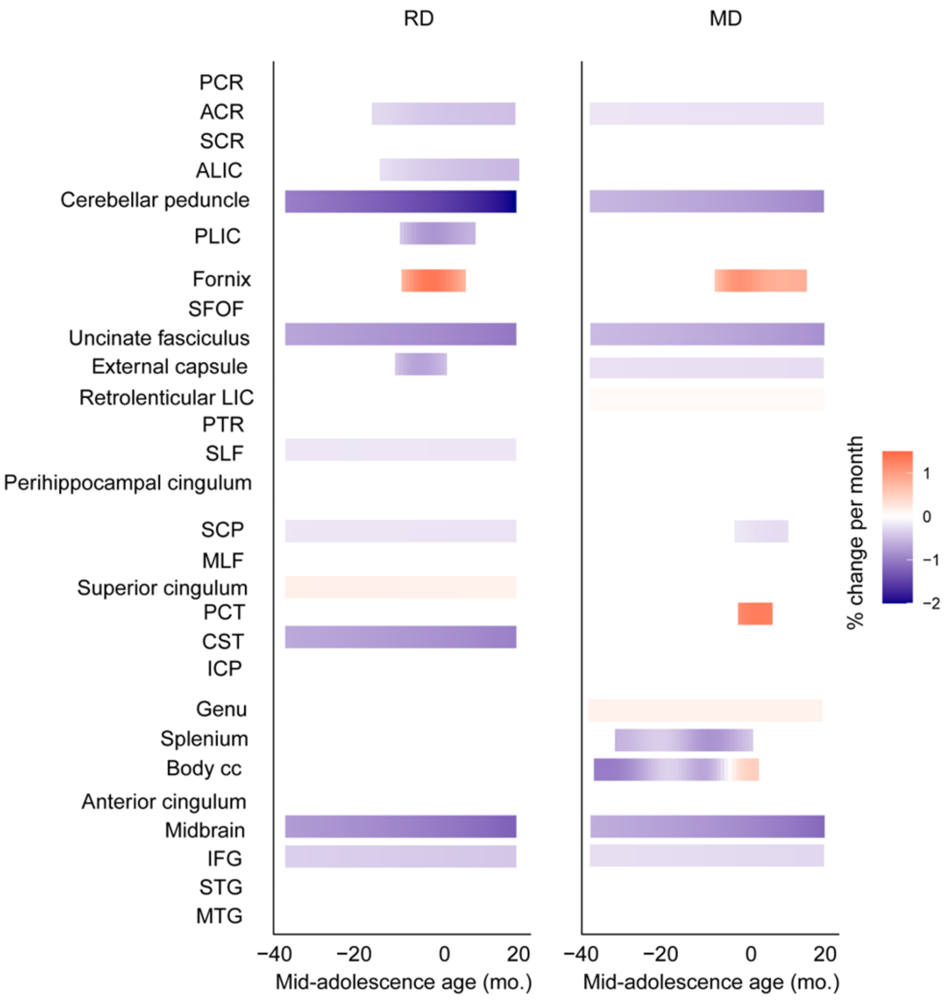

Supplementary Fig. 10. White matter maturation during development. Similar to Figure 5g, Stages of significant growth and timing of maturation of Radial Diffusivity (RD) and Mean Diffusivity (MD) of major white matter tracts. Each row is an ROI grouped to projection, association and commissural tracts, and brainstem white matter regions and short-range white matter. Rows are sorted in the same order as in Figure 5g, according to their time of maturation of FA in each group. Colors represent % change per month (red = increase, blue = decrease).
