# Supplementary material for: Longitudinal measures of monkey brain structure and activity through adolescence predict cognitive maturation
Source: Nat Neurosci. 2025 Oct 27;28(11):2344–55. doi: 10.1038/s41593-025-02076-0 (PMC12586175; doi:10.1038/s41593-025-02076-0)
Supplement: Supplementary file 2 — Reporting Summary [file 41593_2025_2076_MOESM2_ESM.pdf]

nature portfolio

Corresponding author(s): Christos Constantinidis

Last updated by author(s): Aug 18, 2025

Reporting Summary

Nature Portfolio wishes to improve the reproducibility of the work that we publish. This form provides structure for consistency and transparency in reporting. For further information on Nature Portfolio policies, see our Editorial Policies and the Editorial Policy Checklist.

Statistics

For all statistical analyses, confirm that the following items are present in the figure legend, table legend, main text, or Methods section.

n/a	Confirmed	
☐	☒	The exact sample size (n) for each experimental group/condition, given as a discrete number and unit of measurement
☐	☒	A statement on whether measurements were taken from distinct samples or whether the same sample was measured repeatedly
☐	☒	The statistical test(s) used AND whether they are one- or two-sided<br>*Only common tests should be described solely by name; describe more complex techniques in the Methods section.*
☐	☒	A description of all covariates tested
☐	☒	A description of any assumptions or corrections, such as tests of normality and adjustment for multiple comparisons
☐	☒	A full description of the statistical parameters including central tendency (e.g. means) or other basic estimates (e.g. regression coefficient) AND variation (e.g. standard deviation) or associated estimates of uncertainty (e.g. confidence intervals)
☐	☒	For null hypothesis testing, the test statistic (e.g. F, t, r) with confidence intervals, effect sizes, degrees of freedom and P value noted<br>*Give P values as exact values whenever suitable.*
☒	☐	For Bayesian analysis, information on the choice of priors and Markov chain Monte Carlo settings
☒	☐	For hierarchical and complex designs, identification of the appropriate level for tests and full reporting of outcomes
☐	☒	Estimates of effect sizes (e.g. Cohen's d, Pearson's r), indicating how they were calculated

Our web collection on statistics for biologists contains articles on many of the points above.

Software and code

Policy information about availability of computer code

Data collection	The data acquisition system has been described in Meyer et al. (2005). Code has been made available through Github: https://github.com/ChristosLab/WaVE.
Data analysis	Data analysis was implemented in MATLAB. Code and instruction are included in the submission.

For manuscripts utilizing custom algorithms or software that are central to the research but not yet described in published literature, software must be made available to editors and reviewers. We strongly encourage code deposition in a community repository (e.g. GitHub). See the Nature Portfolio guidelines for submitting code & software for further information.

Data

Policy information about availability of data

All manuscripts must include a data availability statement. This statement should provide the following information, where applicable:

- Accession codes, unique identifiers, or web links for publicly available datasets
- A description of any restrictions on data availability
- For clinical datasets or third party data, please ensure that the statement adheres to our policy

Data for the current study are available through this doi:
10.5281/zenodo.14832030

Research involving human participants, their data, or biological material

Policy information about studies with human participants or human data. See also policy information about sex, gender (identity/presentation), and sexual orientation and race, ethnicity and racism.

Reporting on sex and gender	*Use the terms sex (biological attribute) and gender (shaped by social and cultural circumstances) carefully in order to avoid confusing both terms. Indicate if findings apply to only one sex or gender; describe whether sex and gender were considered in study design; whether sex and/or gender was determined based on self-reporting or assigned and methods used. Provide in the source data disaggregated sex and gender data, where this information has been collected, and if consent has been obtained for sharing of individual-level data; provide overall numbers in this Reporting Summary. Please state if this information has not been collected. Report sex- and gender-based analyses where performed, justify reasons for lack of sex- and gender-based analysis.*
Reporting on race, ethnicity, or other socially relevant groupings	*Please specify the socially constructed or socially relevant categorization variable(s) used in your manuscript and explain why they were used. Please note that such variables should not be used as proxies for other socially constructed/relevant variables (for example, race or ethnicity should not be used as a proxy for socioeconomic status). Provide clear definitions of the relevant terms used, how they were provided (by the participants/respondents, the researchers, or third parties), and the method(s) used to classify people into the different categories (e.g. self-report, census or administrative data, social media data, etc.) Please provide details about how you controlled for confounding variables in your analyses.*
Population characteristics	*Describe the covariate-relevant population characteristics of the human research participants (e.g. age, genotypic information, past and current diagnosis and treatment categories). If you filled out the behavioural & social sciences study design questions and have nothing to add here, write "See above."*
Recruitment	*Describe how participants were recruited. Outline any potential self-selection bias or other biases that may be present and how these are likely to impact results.*
Ethics oversight	*Identify the organization(s) that approved the study protocol.*

Note that full information on the approval of the study protocol must also be provided in the manuscript.

Field-specific reporting

Please select the one below that is the best fit for your research. If you are not sure, read the appropriate sections before making your selection.

☒ Life sciences ☐ Behavioural & social sciences ☐ Ecological, evolutionary & environmental sciences

For a reference copy of the document with all sections, see nature.com/documents/nr-reporting-summary-flat.pdf

Life sciences study design

All studies must disclose on these points even when the disclosure is negative.

Sample size	The study included a total of 14 animals. Detailed numbers of sessions, trials, and neurons are reported in the text. No statistical methods were used to pre-determine sample sizes but our sample sizes are similar to those reported in previous publications (23,24).
Data exclusions	Neurons were only excluded if they did not contain a sufficient number of trials (n<4) because estimation of mean firing rate for downstream analysis cannot be done reliably. This requirement was based on similar prior studies (ref. 23, 24).
Replication	Experiments were repeated for each animal and data analysis are conducted across all animals to ensure consistency and reliability of the results. For statistical analysis, bootstrap tests were used. Details of the procedures and consistency of results are provided in the methods section.
Randomization	We distinguish between three cohorts: Cohort A included eight monkeys (6 male, 2 female) and was tracked throughout adolescence. Cohort B included four monkeys (4 male) and was tested at two time points (early adolescence, adulthood), providing a control for training exposure. Cohort C included two monkeys (2 female) that were first trained in adulthood and provided a control for age without training. Group of the monkeys are randomly assigned at the beginning of the study.
Blinding	Data collection was not performed blind to the conditions of the experiments. However, the same analysis pipelines and custom-written code was used for both data collection and analysis to ensure consistency and minimize bias.

Reporting for specific materials, systems and methods

We require information from authors about some types of materials, experimental systems and methods used in many studies. Here, indicate whether each material, system or method listed is relevant to your study. If you are not sure if a list item applies to your research, read the appropriate section before selecting a response.

Materials & experimental systems

n/a	Involved in the study
☒	☐ Antibodies
☒	☐ Eukaryotic cell lines
☒	☐ Palaeontology and archaeology
☐	☒ Animals and other organisms
☒	☐ Clinical data
☒	☐ Dual use research of concern
☒	☐ Plants

Methods

n/a	Involved in the study
☒	☐ ChIP-seq
☒	☐ Flow cytometry
☐	☒ MRI-based neuroimaging

Animals and other research organisms

Policy information about studies involving animals; ARRIVE guidelines recommended for reporting animal research, and Sex and Gender in Research

Laboratory animals	We report this information in the result and method sections: 14 monkeys (10 male and 4 female) rhesus monkeys (Macaca mulatta) from an age of 3.0±0.1 to 7.1±0.1 years were used in this study, obtained from Alpha Genesis, SC.
Wild animals	No wild animals were used
Reporting on sex	Behavioral, imaging, and neurophysiological recordings were obtained from both gender of monkeys (10 male and 4 female).
Field-collected samples	No field collected samples were used
Ethics oversight	The Institutional Animal Care and Use Committees of Wake Forest University and the Institutional Animal Care and Use Committees of Vanderbilt University

Note that full information on the approval of the study protocol must also be provided in the manuscript.

Plants

Seed stocks	*Report on the source of all seed stocks or other plant material used. If applicable, state the seed stock centre and catalogue number. If plant specimens were collected from the field, describe the collection location, date and sampling procedures.*
Novel plant genotypes	*Describe the methods by which all novel plant genotypes were produced. This includes those generated by transgenic approaches, gene editing, chemical/radiation-based mutagenesis and hybridization. For transgenic lines, describe the transformation method, the number of independent lines analyzed and the generation upon which experiments were performed. For gene-edited lines, describe the editor used, the endogenous sequence targeted for editing, the targeting guide RNA sequence (if applicable) and how the editor was applied.*
Authentication	*Describe any authentication procedures for each seed stock used or novel genotype generated. Describe any experiments used to assess the effect of a mutation and, where applicable, how potential secondary effects (e.g. second site T-DNA insertions, mosiacism, off-target gene editing) were examined.*

Magnetic resonance imaging

Experimental design

Design type	Anesthetized resting state
Design specifications	MRIs were collected every 3 months from 2.8 years (34 months) of age to 5.8 years (69 months) of age of the monkeys. In total we collected 84 sessions from 8 monkeys.
Behavioral performance measures	N/A

Acquisition

Imaging type(s)	structural, functional, diffusion
Field strength	3T
Sequence & imaging parameters	T1-weighted MPRAGE sequence: TR = 2700 ms, TE = 3.32 ms, inversion time = 880, FOV = 128 × 128 mm, 192 slices of 0.5 mm thickness, resolution = 0.5 mm isotropic; Resting state multiband EPI sequence: TR = 700 ms, TE = 32.0 ms, flip angle = 52°, repetitions = 700, FOV = 128 × 128 mm, 32 slices, resolution = 2 mm isotropic
Area of acquisition	Whole brain

Diffusion MRI ☒ Used ☐ Not used

Parameters: A diffusion-weighted spin-echo echo-planar imaging sequence was utilized to obtain 82 whole-brain slices of 2mm thickness in 30 directions

Preprocessing

Preprocessing software	We reported in method section: Spatial pre-processing was performed using a pipeline coded in python, which relied on functions from AFNI, ANTs, FSL, FreeFurfer and Connectome Workbench for inhomogeneity correction, spatial and surface registration to a standardize space, the study template space.
Normalization	We reported in method section: The study template was based on the average of the last T1 anatomical image (T1last) of each animal when registered in a common space using the function "anats_to_common" from Sammba-MRI.
Normalization template	We reported in the method section: A high-resolution NMT template (NIH Macaque Template) as well as the CHARM and SARM atlases segmentation were registered to the study template. Individual anatomical T1 images (T1n) were registered to their T1last and each T1last was registered to the study template.
Noise and artifact removal	We reported in the method section: The two movement parameters (T1n to T1last and T1last to study template) were combined to register T1n to the study template. The animals were intubated and artificially ventilated at about 20 breaths per minute. Expired CO2 was monitored and maintained between 35 and 45 mmHg. Animals were scanned under isoflurane anesthesia at 1%–1.5%.
Volume censoring	We reported in the method section: Inversion of these movement parameters was used to register the atlases to the individual T1n images. The co-registrations of the atlases to the T1 were individually inspected and corrected manually when necessary.

Statistical modeling & inference

Model type and settings	We employed Generalized Additive Mixed Models (GAMMs) to analyze the developmental trajectories of volume, thickness, white matter integrity of ROIs. The models included both fixed effects (age) and random effects (individual variability) to account for within-subject correlations. To ensure flexibility and avoid underfitting, we used a sufficiently large number of knots for the smooth terms, allowing the model to capture complex non-linear patterns in the data. The smoothing parameters were selected using restricted maximum likelihood (REML) estimation.
Effect(s) tested	The primary effects tested were the changes in volume, thickness, white matter integrity of ROIs across adolescence.

Specify type of analysis: ☐ Whole brain ☐ ROI-based ☒ Both

Anatomical location(s): We used NMT template (NIH Macaque Template) and used the CHARM atlas level 1 and level 2 to automatically label ROIs. Individual anatomical T1 images (T1n) were registered to their T1last and each T1last was registered to the study template.

Statistic type for inference (See Eklund et al. 2016)	N/A
Correction	N/A

Models & analysis

n/a	Involved in the study
☒	☐ Functional and/or effective connectivity
☒	☐ Graph analysis
☐	☒ Multivariate modeling or predictive analysis

Multivariate modeling and predictive analysis: We employed Generalized Additive Mixed Models (GAMMs), which included multiple predictors (age, individual variability) to analyze the relationships between these variables and the developmental trajectories of ROIs.
